# Functional unknomics of the SAR11 clade reveal hidden genetic potential underlying adaptation to bottom-up and top-down pressures

**DOI:** 10.1128/msystems.01026-26

**Published:** 2026-09-17

**Authors:** Satoshi Nishino, Kento Tominaga, Kimiho Omae, Teppei Deguchi, Koji Hamasaki, Susumu Yoshizawa, Yuki Nishimura

**Affiliations:** 1 Department of Integrated Biosciences, Graduate School of Frontier Sciences, The University of Tokyo 34811, Kashiwa, Chiba, Japan; 2 Atmosphere and Ocean Research Institute, The University of Tokyo 13143, Kashiwa, Chiba, Japan; 3 Department of Natural Environmental Studies, Graduate School of Frontier Sciences, The University of Tokyo 595388, Kashiwa, Chiba, Japan; 4 RIKEN Pioneering Research Institute 13593, Wako, Saitama, Japan; 5 Department of Life Science, College of Science, Rikkyo University 196841, Toshima-ku, Tokyo, Japan; 6 Collaborative Research Institute for Innovative Microbiology, The University of Tokyo, Bunkyo-ku, Tokyo, Japan; The University of Texas at Austin, Austin, Texas, USA

**Keywords:** SAR11, Pelagibacterales, unknome, functionally unknown genes, antiviral defense system

## Abstract

A substantial fraction of the genes in bacteria lack detectable sequence similarity to genes with known functions. These functionally uncharacterized genes—collectively referred to as the “unknome”—represent a largely unexplored genetic repertoire harboring insights into marine bacterial ecology. In this study, we explored the function of the unknome of the SAR11 clade, the most abundant bacterial lineage in the ocean, with a particular focus on genes that provide insight into its ecology. Based on the Clusters of Orthologous Genes and Kyoto Encyclopedia of Genes and Genomes classifications, approximately 56% of SAR11 ortholog groups were classified as members of the unknome. Among the SAR11 unknome, we successfully inferred the functions of 57 ortholog groups that are conserved in the SAR11 clade by protein structure similarity searches and genomic context analyses. These ortholog groups include putative transporter components, supporting the current ecological understanding that the SAR11 clade is specialized in substrate uptake to adapt to oligotrophic marine environments. Furthermore, structural analysis indicated that the DUF2237-containing protein, enriched in marine environments, may interact with purine nucleotide-containing compounds. This may suggest the existence of unique nucleotide utilization mechanisms in marine bacteria. In addition, we identified candidate viral defense systems within the unknome, indicating that diverse defense systems are present in at least one-third of cultured SAR11 strains. The presence of these defense systems, even within streamlined SAR11 genomes, suggests that they confer significant ecological advantages. Our analyses provide insights into the genetic basis of bottom-up processes (adaptation to oligotrophic environments) and top-down processes (antiviral defense strategy) contributing to ecological success.

**IMPORTANCE:**

Many microbial genes have no experimentally established function, limiting our ability to explain how microorganisms adapt to their environments. We examined this uncharacterized gene space, or “unknome” in SAR11, the most abundant bacterial clade in the ocean, by integrating evolutionary conservation, genomic context, predicted protein structure, and environmental distribution. This approach enabled us to prioritize components of the SAR11 unknome, including a core unknome conserved across the clade and genes enriched in specific lineages, and to identify several candidates with possible ecological roles in nutrient acquisition and defense against viruses. Our results suggest that the SAR11 unknome contains important clues to the ecological success of SAR11 rather than merely reflecting incomplete annotation or gene-prediction artifacts. Our study highlights the potential value of unknome analysis for identifying ecologically relevant genes in environmental microorganisms.

## INTRODUCTION

The SAR11 clade, also known as the order *Candidatus* Pelagibacterales, is the most abundant group of heterotrophic bacteria in marine environments, accounting for approximately 25% of all bacterioplankton cells in the ocean photic zone (1). Due to its high abundance, members of the SAR11 clade play crucial roles in marine ecosystems and biogeochemical cycles (2, 3). Notable characteristics of the SAR11 clade include slow growth rates and highly streamlined genomes, which typically lack accessory genes such as antiviral defense systems and, occasionally, essential biosynthetic pathways (3–6). Due to their poor growth under laboratory conditions, culture-based studies of the SAR11 clade are limited in achieving comprehensiveness and throughput, and the mechanisms by which they maintain their ecological dominance are still not fully understood.

The ecological success of the SAR11 clade has been explored from both bottom-up and top-down perspectives. On the bottom-up side, their extremely resource-efficient lifestyle is thought to provide a strong advantage in oligotrophic marine environments. Several observations support this view, including genome streamlining (5, 7), the presence of multifunctional transporters and highly expressed and highly efficient solute-binding proteins (8–10), loss of motility and transcriptional regulation (5, 7, 11), and light utilization by proteorhodopsin (12, 13).

In contrast, from a top-down perspective, SAR11 has historically been discussed as a defense-specialist lineage with relatively low susceptibility to predation and viral lysis in relation to the “kill-the-winner” hypothesis. According to this hypothesis, fast-growing species are expected to be more susceptible to viral infection, and this framework was proposed as one explanation for how slow-growing SAR11 strains can maintain high abundance (14, 15). However, the isolation of Pelagiphages (16) and evidence that they frequently infect SAR11 populations (17–20) suggest that this clade is subject to substantial virus-mediated top-down pressure, challenging such a simplified view. The “king of the mountain” (KoM) hypothesis further suggests that the high abundance of SAR11 promotes frequent genetic recombination within populations, which could influence viral susceptibility (21). The KoM hypothesis is supported by the high intraspecific recombination rate observed in the SAR11 clade (22, 23). Such recombination may contribute to diversification of genomic regions encoding membrane proteins that potentially act as viral receptors, which may help SAR11 populations evade viral infection (24). However, there is still a lack of direct evidence for the presence of antiviral defense systems and their frequent horizontal gene transfer among the SAR11 clade (3, 20).

Currently, genome-based approaches play indispensable roles in microbiology, particularly for lineages that are difficult to cultivate, such as the SAR11 clade. However, a growing challenge in the field is the discovery of a large number of genes with no similarity to any functionally characterized sequences (25, 26). The problem is particularly pronounced in marine metagenomes, where a substantial fraction of predicted genes remain functionally unannotated (25, 27). Recent discoveries of diverse antiviral defense systems encoded by previously uncharacterized genes (28) demonstrate that ecologically important functions can be hidden within this uncharacterized gene space. To overcome the limitation of sequence-based annotation, an increasing number of studies are adopting bioinformatics approaches that go beyond sequence similarity, including protein structure prediction and genomic context analysis (29–31). However, these cutting-edge approaches that could substantially enhance functional annotation have not been systematically applied to the SAR11 clade.

Previous research on genes of unknown function has primarily focused on model organisms (26, 32, 33) or large-scale sequence analyses of whole ecosystems, involving metagenome-assembled genomes (MAGs) and single-amplified genomes (SAGs) (25, 29, 30). Here, we conducted the first systematic investigation of the functionally unknown genes in the SAR11 clade, collectively referred to as the SAR11 functional unknome (26), aiming to infer the functions of conserved genes lacking functional annotation. We placed particular emphasis on genes of ecological importance in the SAR11 clade, including those putatively involved in substrate uptake, phototrophy, and virus-host interactions. By combining structure-based predictions, genomic context information, and environmental distribution patterns, we inferred potential functions for conserved components of the SAR11 unknome. Our results provide new insights into genetic features associated with nutrient acquisition, environmental adaptation, and antiviral defense, highlighting the value of functional unknomics for uncovering ecological mechanisms in non-model marine bacteria.

## MATERIALS AND METHODS

### Collection and evaluation of the SAR11 genomes

We compiled the SAR11 genomes analyzed by Haro-Moreno et al. (34) and expanded the data set with genomes from freshwater, brackish water, Eastern Pacific, and polar environments to better represent the under-sampled clades (Table S1) (35–38). The final data set includes 321 genomes, mainly from cultured strains and SAGs, as MAGs from the SAR11 clade are difficult to reconstruct (39). Genome quality was assessed by using CheckM2 v1.0.2 (Fig. S1) (40). Pelagibacterales genomes with contamination greater than 10% were excluded from the final data set (Table S1).

### Phylogenomic classification of the SAR11 genomes

A concatenated alignment of 120 bacterial single-copy genes (5,036 sites) was constructed using GTDB-Tk v2.1.0 classify-wf (41). Eight genomes were excluded because they had <10% of the alignment sites covered. The *Rickettsia bellii* genome (GCA_002078315.1) served as the outgroup following a previous study (34). The maximum-likelihood tree was inferred with IQ-TREE2 v2.2.0.3 using the ModelFinder-selected Q.insect+F+I+R9 model, with 1,000 ultrafast bootstrap and 1,000 SH-like approximate likelihood ratio test (SH-aLRT) replicates (42, 43). The tree was visualized in R using ggplot2, ggtree, and ggtreeExtra (44–46). Subclades were assigned based on tree topology following the previous classifications (3, 6, 34, 38). Because the phylogenetic positions of subclades IV and V are debated and are not classified within the *Ca*. Pelagibacterales in previous studies (34, 47–50), we treated them separately from subclades I–III in subsequent conserved unknome analyses.

### Functional annotation of ortholog groups and distribution of the SAR11 unknome

Protein-coding genes in 321 genomes (subclades I–V) were predicted with Prokka v1.14.6 (51) and clustered into ortholog groups (OGs) using SonicParanoid2 v2.0.3 with default settings (52). Proteins with at least one predicted transmembrane helix were classified as transmembrane proteins based on TMHMM 2.0 (53), except for those with a single predicted helix within the first 60 amino acids (First60 >10). This exclusion was applied because N-terminal signal peptides can be misclassified as transmembrane helices by TMHMM (53). All coding sequences were annotated with Clusters of Orthologous Genes (COGs) 2020 and Kyoto Encyclopedia of Genes and Genomes (KEGG) Orthology (KO) using COGclassifier v1.0.5 (https://github.com/moshi4/COGclassifier) and KofamScan v1.3.0 (54), respectively, both with default options. Unknome genes were defined as genes lacking functional annotation in both COG and KO, including those not annotated by COGclassifier or assigned to COG categories S (unknown function) or R (general predicted only), and either not assigned to any KO entry or annotated as hypothetical proteins; unknome OGs were those with >50% unknome members. The OG distributions among each subclade were visualized with UpSetR (55).

The SAR11 conserved unknome was defined as unknome OGs occurring in more than 100 genomes and present in all three SAR11 clades (I–III). Environmental distribution data of the conserved unknome and bacteriorhodopsin were retrieved from the Global Microbial Gene Catalog (GMGC) v1.0 using their COG IDs (56). We analyzed GMGC entries with both COG annotations and habitat/source information. Habitat proportions were calculated per OG, counting multiple habitat annotations independently. To ensure sufficient representation, we focused on “marine,” “soil,” and “human gut” samples, grouping all other environments as “others.”

### Neighborhood OG analysis

Genomic contexts of all coding sequences were analyzed using GFF files generated by the Prokka pipeline. Two genes were considered neighbors if located within five genes on the same contig. Neighboring gene pairs were aggregated across genomes based on the corresponding OGs. Pairs were retained when the same co-localization pattern was observed in ≥75% of genomes in which both OGs co-occurred (minimum of 10 genomes). The network was visualized using igraph (57) and ggnetwork (58). To reduce false positives arising from phylogenetic signal in this closely related lineage, we applied more stringent thresholds than previous studies (29). In addition, because extensive core genome conservation within the SAR11 clade can complicate the interpretation of co-occurrence patterns (6), subclades V (the HIMB59 clade) and IV were retained as comparative groups in this analysis. Their inclusion provided additional phylogenetic contrast, facilitating the identification of co-occurrence patterns that are less likely to arise solely from shared evolutionary history. Pairs with highly unequal occurrence frequencies may not meet this criterion, even if the rarer OG always co-occurs with the more prevalent one. Neighboring pairs containing the OG1814 or OG1249 were manually checked using AlphaFold2, and complex formation was evaluated by pDockQ and pDockQ2 scores (59–61).

### Collection and comparison of predicted protein structures

All protein structures of *Ca*. Pelagibacter ubiqueversans, formerly known as *Ca*. P. ubique, HTCC1062 strain (subclade Ia.1) and *Ca*. Pelagibacter sp. IMCC9063 strain (subclade IIIa.3) were downloaded from the AlphaFold Protein Structure Database (62). To improve the structural prediction of the conserved unknome OGs, the structures of the conserved unknome proteins of the HTCC1062 strain were predicted with AlphaFold2 after excluding their signal peptide regions predicted by DeepTMHMM version 1.0.24 (63). Structural similarity searches for the unknome proteins were performed using Foldseek version 7.04e0ec8 (64) against the Protein Data Bank (PDB) downloaded on 4 September 2023 (65).

### Metatranscriptome data analysis of SAR11 genes

Sequence reads of the 509 metatranscriptomes obtained from the Tara Oceans samples were mapped onto the 321 SAR11 genomes (Table S2) (66). We downloaded paired-end sequence data from NCBI SRA using the SRA toolkit (https://github.com/ncbi/sra-tools), and then quality control was performed using fastp with options -q 20 -n 10 -l 60 (67). The quality-controlled reads were mapped via bwa-mem2 (68). Transcripts per million were calculated using a script from a previous study (https://github.com/yosuken/CountMappedReads2) (69).

### Analysis of the light-related uncharacterized protein DUF2237

A total of 7,968 DUF2237 domain-containing proteins were retrieved from the Pfam database on 21 April 2023. These sequences were annotated with domain information using pfam_scan (70) and aligned using MAFFT v7.508 (71) in auto mode. The alignment was visualized on the predicted monomer structure of the DUF2237-containing protein of the HTCC1062 strain using ConSurf-DB (72) and PyMOL v2.5.4 (73). The Predicted Aligned Error plot was visualized using a Python package (https://github.com/cddlab/alphafold3_tools).

As previous studies have suggested a potential association between DUF2237 and proteorhodopsin (74), we included proteorhodopsin in this analysis to assess their phylogenetic co-distribution. The OceanDNA MAG catalog v1 (75) and the Pfam database were utilized to assess the phylogenetic distribution of DUF2237 genes. Genes annotated as COG3651 and COG5524 were identified as DUF2237-containing proteins and proteorhodopsin, respectively. A multiple sequence alignment of 120 marker genes from 7,696 representative MAGs in the OceanDNA MAG catalog was generated with the GTDB-Tk classify-wf. The resulting alignment contained 5,036 sites. Subsequently, a maximum likelihood phylogenetic tree was inferred with IQ-TREE2 (v2.2.0.3) using the ModelFinder-selected Q.yeast+R10 model, with 1,000 SH-aLRT replicates. The phylogenetic distribution of DUF2237 and proteorhodopsin was visualized on the phylogenetic tree using ggplot2, ggtree, and ggtreeExtra (44–46).

The structure of the HTCC1062 strain’s DUF2237 protein, predicted by AlphaFold2, was prepared in PDBQT format using MGL Tools v1.5.7 (76) for docking simulations. A ligand library was constructed by converting 258 compounds with biological roles from the KEGG Compound database (br8001) and 7 nucleotide-containing molecules from the Protein Data Bank Japan (NAI, PO4, NDP, FAD, COA, C2E, and NAD) into PDBQT format using Open Babel v3.1.0 (77) and MGL Tools (Table S3). The grid box was defined as 25 × 20 × 20 Å around the pocket site of the DUF2237 protein predicted by PrankWeb 3 (78). All docking simulations were performed using AutoDock Vina v1.2.5 (79), with an exhaustiveness value of 100. The possibility of binding to ATP was evaluated using ATPbind (80).

### Defense system annotation

DefenseFinder (updated on 5 June 2024) was used to identify potential antiviral defense genes (81). To check for the completeness of defense systems, neighboring genes of each defense gene found by DefenseFinder were manually annotated and compared to known defense genes registered to the DefenseFinder wiki (82) by genomic context, sequence alignment using MAFFT, and structure similarity search using AlphaFold2 and Foldseek.

## RESULTS

### Collection and phylogenomic analysis of SAR11 genomes

**Fig 1:**
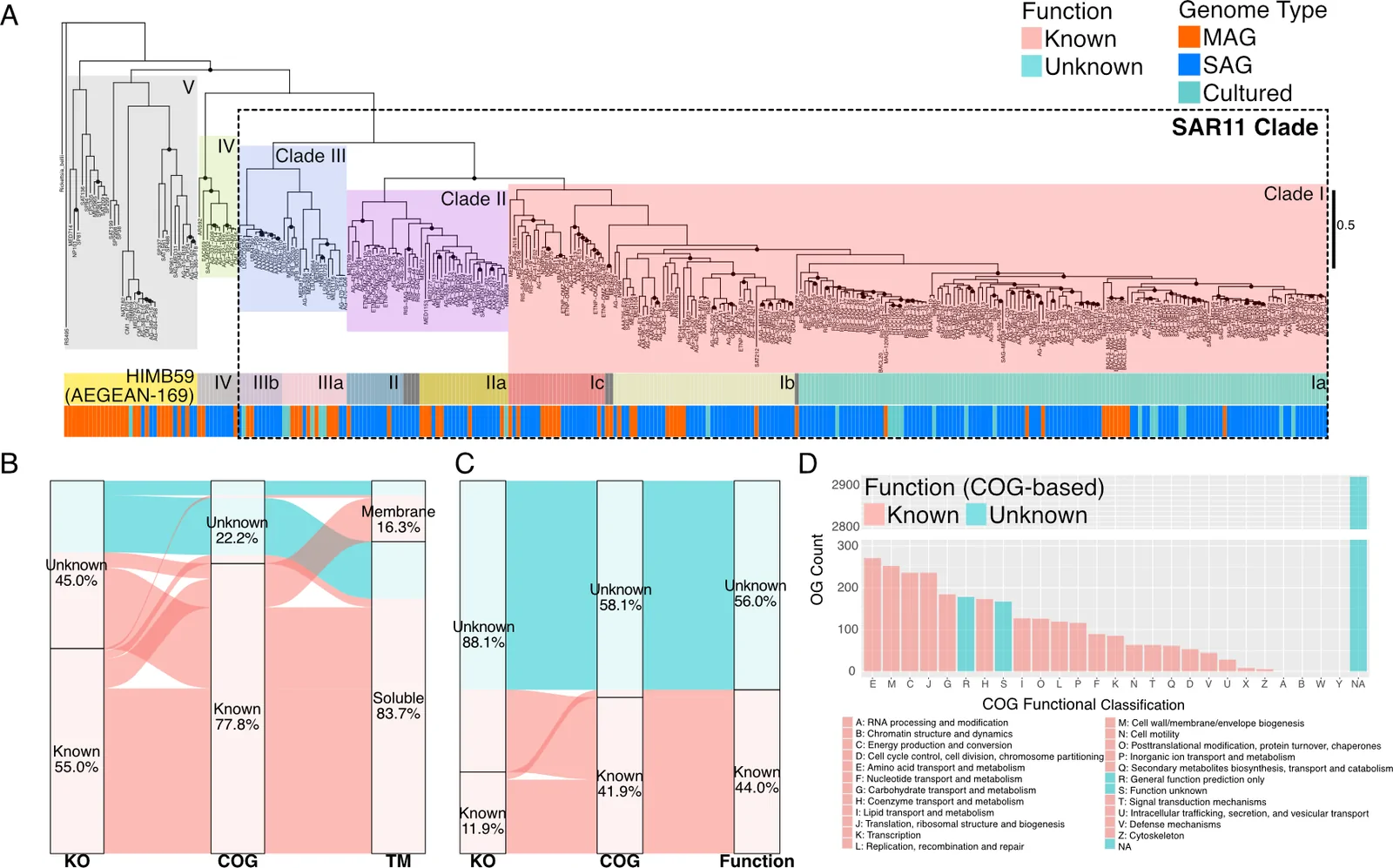
Characterization of the unknome from a phylogenetically comprehensive SAR11 genome data set. (**A**) Maximum-likelihood phylogenetic tree of the SAR11 and related clade genomes. The color coding in the phylogenetic tree indicates five subclade classifications. The colors below provide more detailed subclade classifications and types of the genomes, that is, MAG (red), SAG (blue), or pure culture (light blue). The dots on the node show UltraFast Bootstrap values >95%. (**B and C**) Sankey plots summarize the annotation profiles of all genes/OGs and transmembrane region prediction results. Blue shows the unknome genes/OGs. TM indicates transmembrane proteins predicted using TMHMM 2.0 (see Materials and Methods for details). (**D**) COG annotation results for all OGs. Blue bars show unknome genes in this study.

We collected 321 SAR11 and related clade genomes (Fig. 1A; Table S1) representing five clades. Among the 306,416 protein-coding genes, 55.0% and 77.8% of genes were assigned to KO and COG annotations, respectively (Fig. 1B). A total of 297,495 (97.1%) protein-coding genes were clustered into 5,603 OGs, of which 11.9% and 41.9% were assigned KO and COG annotations, respectively (Fig. 1C and D; Table S4). The lower annotation rate is not unexpected, given differences in database coverage and annotation methodologies between KO and COG, including the profile-specific thresholds used by KofamScan (54).

We classified OGs according to their conservation across the five clades. The largest set combination corresponded to OGs conserved across all subclades (968 OGs), exceeding those unique to any single subclade (Fig. S2A). This pattern is consistent with the streamlined SAR11 genomes, in which the core genome constitutes a large proportion (5, 6). Among the COG-assigned genes, categories E (amino acid transport and metabolism, 271 OGs), M (cell wall/membrane/envelope biogenesis, 252 OGs), C (energy production and conversion, 236 OGs), and J (translation, ribosomal structure and biogenesis, 236 OGs) were the most frequently observed in the SAR11 clade (Fig. 1D). The ordering of these categories, such as category M exceeding category K (transcription), is consistent with reduced transcriptional regulation, a feature of genome streamlining (5–7).

### Definition and characteristics of the “unknome”

By defining unknome genes as those that could not be functionally annotated by either KO or COG, 56.0% of OGs were classified as members of the unknome (Fig. 1D). The unknome genes were shorter and more AT-rich than functionally annotated genes (Fig. S3A and B). As in *Escherichia coli* unknome (32), SAR11 unknome genes showed a slightly higher proportion of TM proteins than functionally known genes (Fig. 1B, Fisher’s exact test, *P* < 2.2e−16). Tara Oceans metatranscriptomes confirmed that unknome OGs were expressed *in situ*, although their expression levels were slightly lower than those of functionally known genes (Fig. S3 and S4, Wilcoxon rank sum test, *P* < 2.2e−16). Most OGs with unknown functions were specific to clade I or the HIMB59 clade (781 and 534 OGs; Fig. S2B), reflecting the high diversity of clade I and the large genome size of the HIMB59 clade (50).

Notably, 191 unknome OGs were specific to clade III, a lineage adapted to low-salinity environments (Fig. S2B). Several contain features suggestive of environmentally relevant functions. For example, OG1777 encodes a predicted EF-hand domain-containing protein that may participate in calcium-dependent environmental sensing (83), whereas OG3994 encodes a DUF1468 family protein associated with a tripartite tricarboxylate transporter (TctABC) gene cluster. OG3994 is distinct from marine TctB homologs (OG1022), sharing at most 30.77% amino acid identity. The marked sequence divergence suggests substantial evolutionary differentiation of this transporter component. Overall, the restriction of these genes to freshwater and brackish lineages suggests that a subset of the unknome may contribute to freshwater adaptation and ecological specialization.

### Functional prediction of the unknome based on neighboring gene functions and predicted protein structures

**Fig 2:**
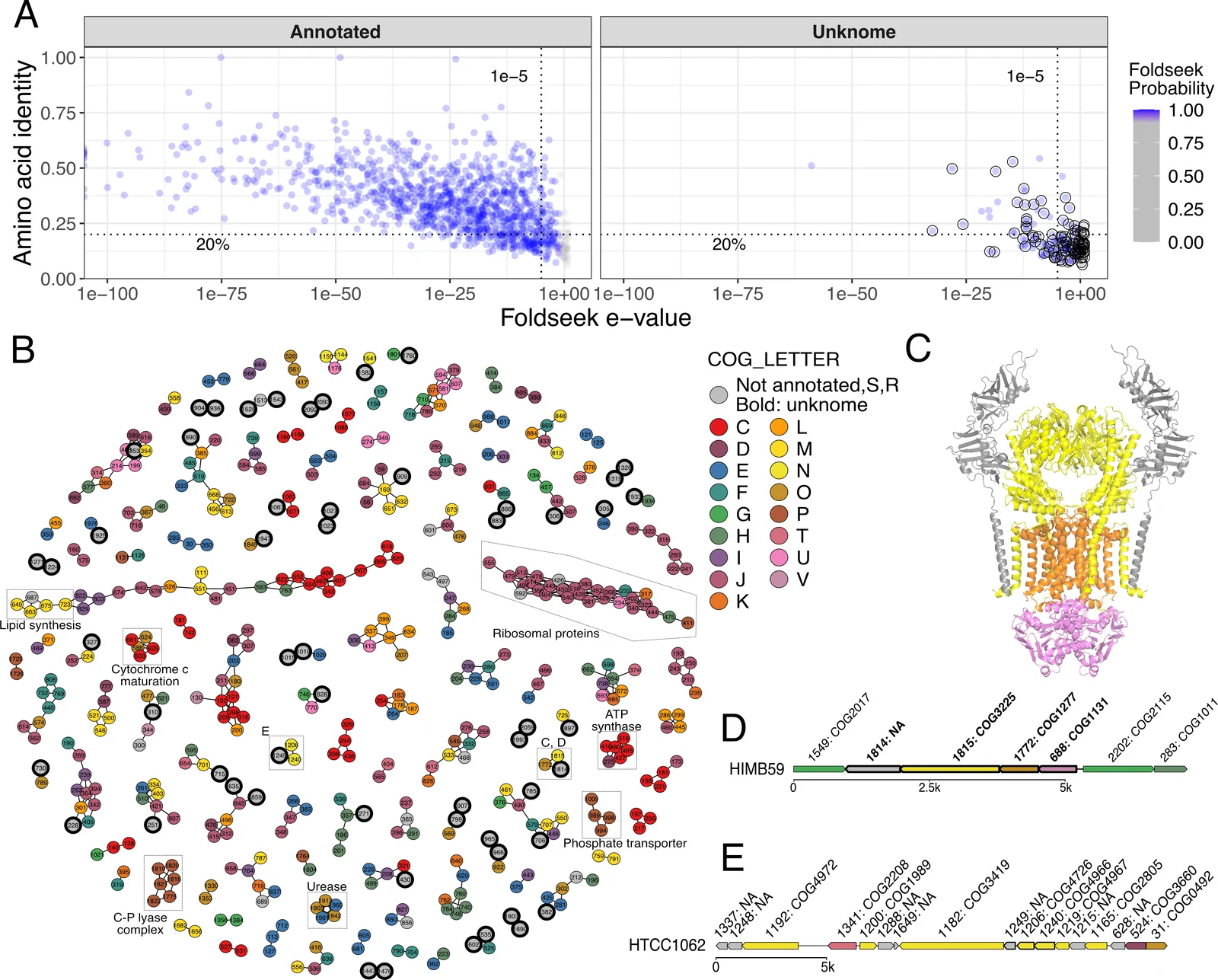
Function prediction based on genomic context and protein structural similarity search. (**A**) Scatter plots of all *Ca*. P. ubiqueversans HTCC1062 proteins compared with known protein structures in the Protein Data Bank. Dot colors indicate the Foldseek probability, and conserved unknome proteins are highlighted with outer black circles. Dotted lines represent thresholds of *e*-value = 1e−5 and amino acid identity = 20%, indicating potential remote homologs. (**B**) Network nodes represent each OG, and colors indicate the COG functional categories. Pairs with a co-localization probability ≧75% within five genes upstream or downstream on the same contig are connected by edges. Several known operons are highlighted with dashed outlines as examples. Unknome OGs are highlighted with thick black outlines. (**C**) Predicted protein complex structure of OG688, OG1772, OG1815, and OG1814 from HIMB59 strain using AlphaFold-Multimer. The complex forms an octameric assembly composed of four homodimers. The protein colors show the COG functional category. (**D and E**) Genome context around each network component in panel **B**. The genes outlined in bold were used for protein structure prediction in panel **C**.

We predicted the functions of the unknome using protein structure prediction and structure similarity searches. Using *Ca*. P. ubiqueversans HTCC1062 (subclade Ia.1) and *Ca*. Pelagibacter sp. IMCC9063 (subclade IIIa) as representatives, we compared predicted protein structures to those in the PDB. In HTCC1062 strain, 1,212/1,328 proteins (91%) showed >90% probability of being homologous to a PDB entry, and 237 of these shared <20% sequence identity with their best hits (Fig. 2A). In IMCC9063 strain, 1,216/1,382 proteins (88%) showed high probability of structural homologs, with 221 of them having <20% sequence identity (Fig. S5). Among unknome proteins, structurally similar proteins were found for 113/190 (59.5%) in HTCC1062 and 120/236 (50.8%) in IMCC9063. These results suggest that structural similarity searches effectively detect potential remote homologs within the SAR11 genomes.

We next applied neighborhood gene analysis, which predicts gene function from the genomic context because functionally related genes often occur in close proximity in prokaryotic genomes (e.g., in operons or syntenic regions) (84, 85). Neighboring OG pairs were defined as genes located within five genes of each other and retained only when showing reciprocal co-localization in at least 75% of genomes in which both OGs co-occurred (see Materials and Methods). We then constructed a neighboring OG network containing 474 OGs and 620 edges that met the neighboring pair criteria (Fig. 2B; Table S6). Functionally related OGs formed connected components, the largest of which corresponded to the ribosomal protein operon.

To explore functional associations, we examined connected components containing both unknome (bold nodes) and functionally annotated OGs. In total, 31 such components were identified among 111 components. For example, OG1814, a DUF4340-containing OG without a COG assignment, was conserved near the genes annotated as ABC transporter components, such as OG1815, OG1772, and OG688. In the HIMB59 strain (GCA_000299115.1), proteins from these OGs were predicted to form an ABC transporter-like complex (Fig. 2C and D; Fig. S6A and B). pDockQ2 estimates the reliability of predicted protein–protein interfaces, supporting the hypothesis that OG1814 functions as a novel transporter subunit (pDockQ2 >0.268). Similarly, OG1249 (COG4696, a DUF2139-containing gene) was conserved within the type IV pilus operon, indicating a potential role as a minor pilus component (Fig. 2E), which may also be associated with the uptake of exogenous nucleic acids (86).

### Function prediction of the conserved unknome OGs

Given that OGs widely conserved throughout the SAR11 clade likely play critical roles in their ecology, we further focused on the 108 SAR11 conserved unknome OGs that are conserved across all three SAR11 clades (I–III). By integrating information from neighboring genes and structurally similar proteins, 75 of the 108 OGs had at least one line of supporting information (Fig. S7). However, in many cases, the functions of the structurally similar proteins or neighboring genes were also functionally uncharacterized (Supplemental text). Then, we performed an extensive literature search using gene and domain names to capture functional information not yet incorporated into current databases. This search identified functional insights for some OGs. Overall, we inferred putative function or obtained additional functional information for 57 of 108 conserved unknome OGs, which was not captured by COG2020 or KEGG annotations (Table S5; Supplemental text). These include predicted components of transporters, channels, or metabolic enzymes (e.g., OG228, OG305, OG814, OG824, OG959, OG969, and OG1036), which may support the SAR11 clade’s adaptation to nutrient-limited environments (Fig. S8; Supplemental text).

### Functional analysis of DUF2237, the unknown gene with a skewed distribution in the ocean

**Fig 3:**
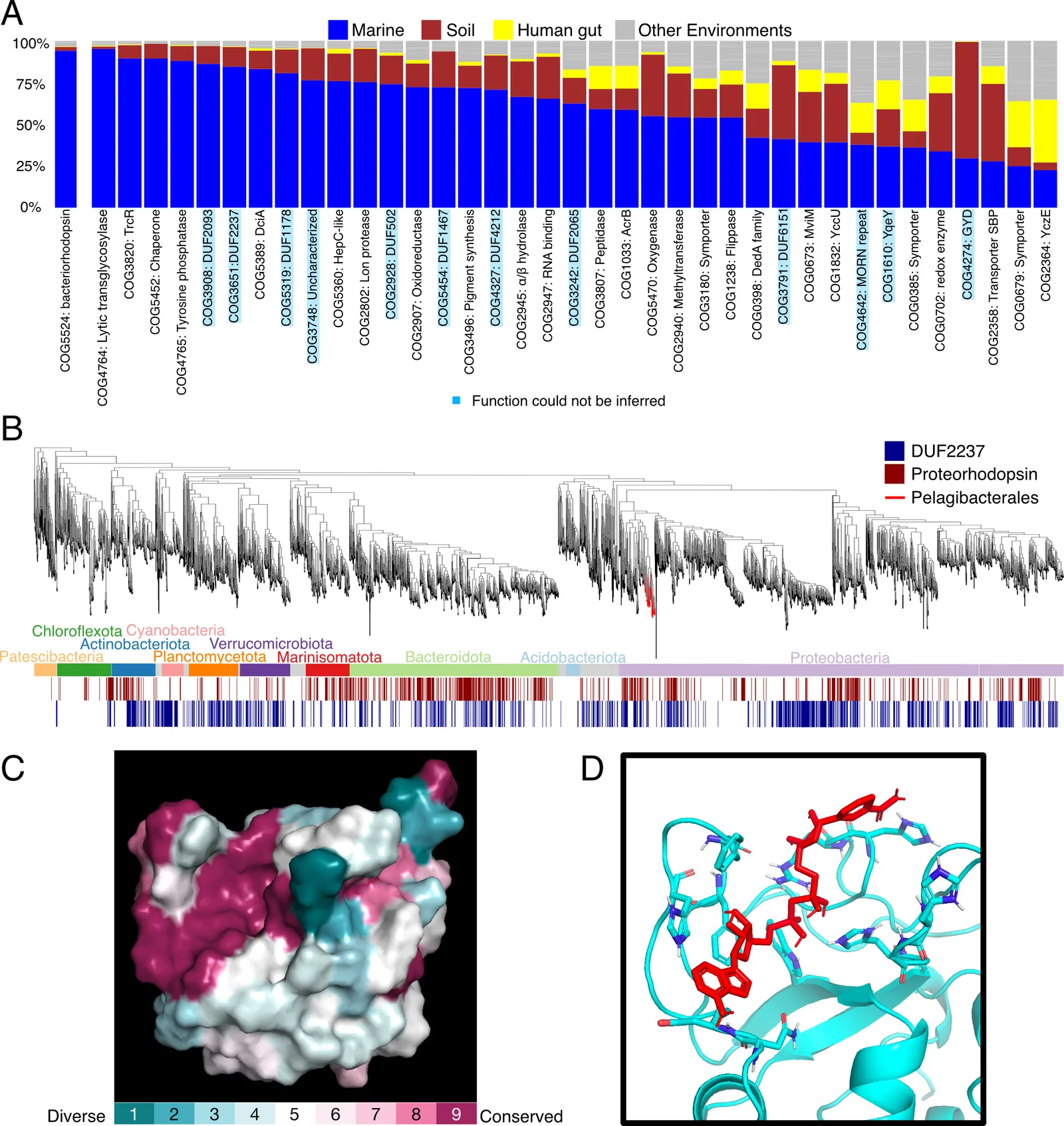
Distribution of the conserved unknome and functional analysis of DUF2237-containing protein. (**A**) Environmental distribution of bacteriorhodopsin (COG5524) and COG-assigned conserved unknome OGs based on GMGC. Bar plot colors indicate habitat, such as marine (blue), soil (brown), and human gut (yellow), and the COG ID and predicted function color indicate whether function could be inferred using the bioinformatics approaches. (**B**) Phylogenetic distribution of the DUF2237-containing gene and proteorhodopsin genes on representative bacterial genomes in the OceanDNA MAG catalog. The heatmap below shows the top 10 phyla and the presence of the DUF2237-containing gene (blue) and the proteorhodopsin gene (brown). Red branches indicate *Ca*. Pelagibacterales. (**C**) The amino acid conservation of 7,968 DUF2237-containing genes in the Pfam database, mapped onto the predicted structure of the DUF2237 protein from *Ca*. P. ubiqueversans HTCC1062. Conservation is color-coded from blue (<10%) to white (50%) to purple (>90%) based on the multiple sequence alignment. (**D**) Top docking pose of *Ca*. P. ubiqueversans HTCC1062 DUF2237 protein (light blue) and NADH (red).

We next examined ecological distribution to infer potential functions of the conserved unknome OGs lacking functional information from any of the above analyses. We focused on the OGs enriched in marine environments, as such genes may provide clues to ecological features associated with the marine adaptation of the SAR11 clade. A representative example is microbial rhodopsin (COG5524: bacteriorhodopsin) (Fig. 3A), which functions as a photoreceptor capturing light energy in oligotrophic waters (87). The most marine-biased unknome OG was COG3908, which contains the DUF2093 domain (Fig. 3A). Although no further functional information was obtained, this OG was consistently located near the lipid synthesis operon in the SAR11 clade (Supplemental text).

The second most marine-biased unknome OG was annotated as COG3651, which contains the DUF2237 domain. In marine *Flavobacteriia*, the phylogenetic distribution of DUF2237-containing genes has been reported to correlate with that of the proteorhodopsin gene, which encodes a light-driven proton pump (74), although the two genes are not necessarily genomically adjacent. In addition, a DUF2237 knockout in *Synechocystis* sp. PCC6803-P showed reduced phototaxis, suggesting a possible link to light-dependent cellular processes (74). Although three different structures of the DUF2237 protein had been experimentally solved (PDB 2LQ3, 3USH, and 4IOH), the mechanistic link to phototaxis remains unclear. Given the ecological importance of light utilization in the SAR11 clade (11, 13, 88), we further analyzed this unknown protein.

Initially, we re-evaluated the association between proteorhodopsin and DUF2237-containing genes using the OceanDNA MAG catalog (75) to validate the previous observation in marine *Flavobacteriia*. As in the previous study, DUF2237 genes and proteorhodopsin were significantly co-present within the same genomes (Fisher’s exact test, *P* < 2.2e−16). The high conservation of DUF2237 genes across most marine bacterial lineages, except the phyla *Patescibacteria* and *Chloroflexota* (Fig. 3B), indicates their broad ecological importance.

The predicted structure of the DUF2237-containing protein from the HTCC1062 strain suggested a possible dimeric assembly (Fig. S9A and B, pDockQ score = 0.314). Analysis of 7,968 DUF2237 domain-containing proteins in the Pfam database showed that this domain typically occurs as a single-domain protein (Fig. S10A and B). Mapping sequence conservation from these Pfam entries onto the structure revealed a highly conserved pocket region, likely a concave ligand-binding site (Fig. 3C). This suggests a potential role for DUF2237 in interacting with a specific ligand.

To identify potential ligands for the DUF2237 pocket, we performed docking simulations using KEGG “Compounds with biological roles,” excluding macromolecules. Nucleotide-containing ligands, including NADH, cyclic di-GMP (c-di-GMP), and cyclic AMP (cAMP), showed the highest affinities for the DUF2237 protein (Fig. 3D; Table S3). ATP-binding prediction also supported nucleotide interaction (80) (probability = 0.85). Because NADH and ATP participate in central metabolic pathways, and c-di-GMP and cAMP act as motility-related second messengers (89–92), these results suggest that DUF2237 may be involved in nucleotide-associated cellular signaling rather than direct light perception. Consistently, cultured SAR11 strains lack motility (11), and DUF2237-encoding genes occur in conserved genomic neighborhoods enriched in redox metabolism and cofactor biosynthesis genes (Fig. S11), further supporting a metabolic or signaling role for DUF2237.

### Antiviral defense genes within the SAR11 unknome

**Fig 4:**
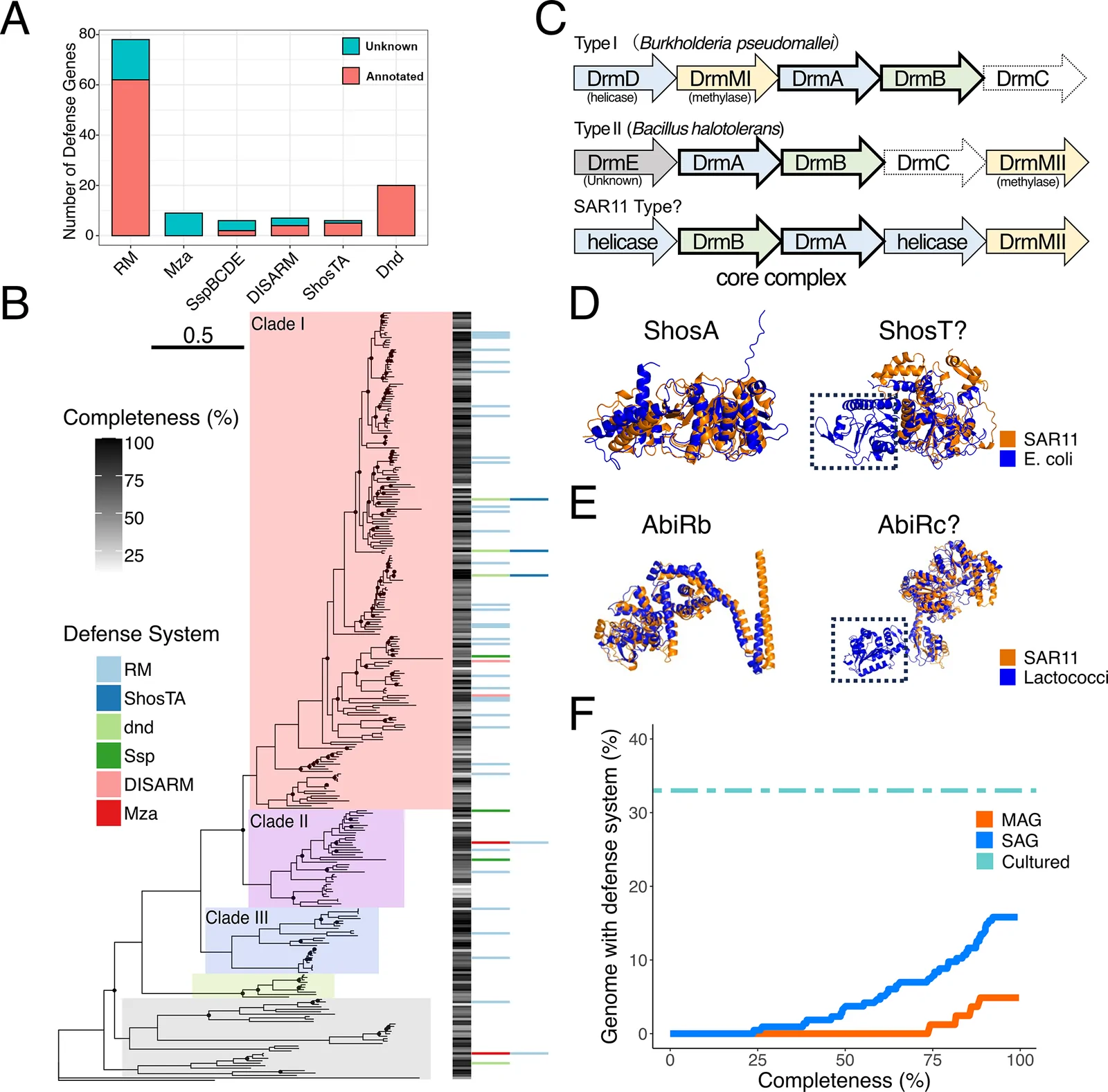
Defense genes identified in SAR11 genomes. (**A**) Bar charts show defense gene counts identified in this study. Blue bars show the unknome genes, and pink bars show annotated genes. (**B**) Sporadic distribution of defense systems. Heatmap colors indicate genome completeness and presence of each defense system. (**C**) Operon structure of known DISARM systems and AG-447-N21 coding DISARM-like system. The colors indicate function as follows: helicase (blue), methylase (yellow), DUF1998-containing protein (green), unknown (gray), and PLD domain (white; not required for defense). (**D**) Predicted structure comparison of the known ShosTA proteins from DefenseFinder wiki (blue) and the ShosTA-like proteins in HTCC1002 (orange). Domain loss is highlighted by the dotted box. (**E**) Predicted structure comparison of known AbiRbc proteins from DefenseFinder wiki (blue) and AbiRbc-like proteins in AG-313-A04 (orange). (**F**) The cumulative proportion of genomes with detected viral defense systems is plotted against genome completeness. The cumulative proportion at each completeness value represents the fraction of genomes at or below that completeness in which at least one defense system (from panel **B**) was detected. Solid blue and red lines show the cumulative proportion for SAGs and MAGs, respectively. The light blue dashed line indicates the detection rate in 24 cultured genomes, serving as a reference.

Recent studies have suggested that virus–host interactions are a major force shaping genomic variation in the SAR11 clade (21, 24, 93). Because antiviral defense systems play a key role in these interactions and are frequently encoded by previously uncharacterized genes (28), we next investigated whether the SAR11 unknome contains genes involved in antiviral defense. Using DefenseFinder (81), we identified a total of 43 defense system occurrences, including restriction-modification (RM) and toxin-antitoxin systems (Table S7). Focusing on the unknome fraction, 21 unknome genes were associated with these defense systems (Fig. 4A). However, because SAR11 genomes are highly divergent (5), defense genes in this lineage may differ substantially from currently characterized systems, potentially limiting the sensitivity of automated detection methods. To identify defense genes that may have been missed by automated methods, we examined genomic regions containing incomplete components of known defense systems and manually curated neighboring genes, as defense genes are often organized in genomic clusters. This extended search identified seven additional putative defense systems, bringing the total to 50 system occurrences across 46 genomes (Fig. 4B). Notably, the Mza, SspBCDE, DISARM, and ShosTA systems have not previously been reported in the SAR11 clade.

Among these newly identified systems, Ssp-like operons were detected in two SAR11 genomes (Fig. 4B). Ssp is a phosphorothioation (PT)-dependent system in which SspBCD phosphorothioates the host bacterial genome and SspE cleaves unmodified foreign ssDNA (94, 95). SAR11 Ssp-like operons encode proteins structurally similar to the known SspBCD (Fig. S12) and an uncharacterized protein shorter than the known SspE. Mza defends against ssDNA phages, although its mechanism remains unknown (96). The DISARM-like operon encodes DrmAB, a DrMII-type methyltransferase, and helicases (Fig. 4C) but lacks DrmC, which is nonessential for defense (97). The distinct gene order suggests a divergent DISARM-like system. The ShosTA toxin-antitoxin system was detected in three SAR11 genomes. The SAR11 ShosT protein is approximately 100 amino acids shorter than the *E. coli* ShosT but retains the phosphoribosyl transferase domain (Fig. 4D), suggesting a truncated but potentially functional toxin, possibly reflecting genome reduction. AbiR is typically encoded by a three-gene operon comprising AbiRa, AbiRb, and AbiRc. AbiRb- and AbiRc-like genes were also present as operons, but the AbiRc-like gene lacks its terminal domain (Fig. 4E). Moreover, although AbiRa is a sigma70 factor, the only sigma70-type RNA polymerase subunit encoded in the SAR11 genome is located elsewhere and not within this operon. Therefore, the functional assignment of this system remains uncertain, and we did not include it as a defense system found in this study.

A previous study showed that only a few deep-sea SAR11 genomes possess part of the CRISPR-Cas-like gene set (98). As in the previous study, Cas-like genes without a CRISPR array were detected in two deep-sea SAGs (AG-337-I02 and AG-410-B18). Three cultured-strain genomes and one MAG possessed *dnd*, a dsDNA PT-based defense system (99). Together with the presence of Ssp-like operons, the detection of dnd suggests that PT-based DNA modifications may contribute to self–nonself discrimination in the SAR11 clade.

Given that defense systems often vary even among closely related strains and are frequently associated with genomic islands (100), these loci may be particularly prone to fragmentation or omission during metagenomic assembly, leading to their underrepresentation in MAGs. To evaluate this possibility, we compared the prevalence of defense systems among cultured genomes, SAGs, and MAGs. Focusing only on high-quality genomes (CheckM2 completeness > 90% and contamination < 5%), defense systems were found in at least one-third of the cultured strain genomes (8/24) and one-quarter of the SAGs (5/20). In contrast, defense systems were rarely detected in MAGs (Fig. 4F). This result suggests that the SAR11 clade harbors more diverse and abundant defense systems than previously inferred from the MAG-based analyses (20).

## DISCUSSION

In the SAR11 clade, a large fraction of OGs remains functionally uncharacterized despite their crucial roles in marine microbial systems. To address this issue, we explored SAR11 unknome OGs using genome context and structural comparison analyses. These unknome OGs include many transporter-related genes (Fig. 2C through E; Fig. S8; Supplemental text), consistent with the substrate uptake strategy of the SAR11 clade (3, 9, 10). Structural similarity searches were particularly effective for annotation (Fig. 2; Fig. S7 and S8; Supplemental text), reflecting extensive sequence divergence and genomic features such as reduced GC content that may hinder homology-based detection (5). These results highlight the value of integrating genomic context and structural prediction for non-model organisms.

Among the remaining unknome genes, we further examined marine-associated candidates that may be ecologically relevant in the SAR11 clade (Fig. 3A; Supplemental text). Among them, DUF2237-containing proteins, previously linked to light-associated phenotypes (74), were further analyzed. Structural modeling and docking simulations suggested potential interaction with purine nucleotide-containing compounds (Fig. 3C and D), including second messengers such as c-di-GMP and cAMP, as well as NADH. These observations suggest that DUF2237 may be involved in fundamental cellular processes not limited to phototaxis. Although definitive conclusions require further experimental validation, the predicted nucleotide-binding property suggests that DUF2237 may represent a previously uncharacterized nucleotide-interacting protein.

We explored the unknome genes to identify viral defense systems that may offer insights into top-down factors contributing to the ecological success of the SAR11 clade. Previous studies suggested that SAR11 possesses only limited viral defense capacity (3, 20). However, our comprehensive genomic analysis revealed a scattered distribution of diverse viral defense systems (Fig. 4A and B). This pattern is consistent with previously reported high intraspecific recombination rates of SAR11 genes (22). While the previous hypothesis proposed that the slow growth rate of SAR11 made it less vulnerable to phage lysis (15), the broader repertoire of defense systems identified in this study suggests ongoing viral pressure, requiring more active resistance to viral infection than previously appreciated. The recent discovery of anti-RM systems in the Pelagiphage genome further supports the arms race between *Ca*. Pelagibacterales and Pelagiphages (82). The presence of defense systems may provide a possible explanation for the ribosome-deprived “zombie cell” phenotype observed during SAR11 viral infection, possibly resulting from abortive infection (20). Moreover, the discovery of the Ssp and Mza systems in the SAR11 genomes, both of which are known to target ssDNA viruses, suggests the existence of undiscovered ssDNA Pelagiphages. Given the high intraspecific recombination rate of SAR11 genes (22), the sporadic distribution of defense systems may reflect population-level sharing of this repertoire rather than frequent transfer across distantly related taxa. Such population-level sharing may help SAR11 balance the trade-off between genome streamlining and defense gene acquisition. This interpretation is consistent with the concept of a bacterial pan-immune system, in which defense genes are frequently gained and lost among closely related strains (100) and also consistent with the KoM hypothesis, which proposes SAR11 maintains its dominance through high diversity and efficient horizontal gene transfer driven by high contact frequency of closely related strains (16, 21). Collectively, these observations suggest a complex evolutionary interplay between SAR11 and its phages, likely shaped by genome streamlining, horizontal gene transfer, and diverse defense strategies. Together, these findings highlight that the SAR11 unknome contributes to both bottom-up processes linked to oligotrophic adaptation and top-down processes driven by antiviral defense, thereby shaping ecological adaptation in marine environments.

Our results also underscore the importance of genome reconstruction methods in evaluating the distribution of viral defense systems. Compared with SAGs and genomes from cultured strains, viral defense genes were underrepresented in MAGs (Fig. 4F), which may have led to the previous observation that SAR11 possesses little viral defense capacity (3, 20). This underrepresentation likely stems from limitations in MAG assembly and binning, reflecting the high variability of specific genomic regions (e.g., hypervariable regions and metagenomic islands) (6, 23, 24). Consequently, SAG analysis and cultivation efforts provide powerful approaches to capture diversity of defense systems, as it circumvents the challenges associated with MAG construction (39).

Despite the insights obtained in this study, several limitations should be acknowledged. Functional assignments here were inferred only from *in silico* analyses and therefore require experimental validation. In addition, the ecological roles of the identified genes and defense systems remain to be experimentally tested in cultured SAR11 representatives. The neighborhood-based network analysis relies on relatively stringent thresholds to reduce spurious gene associations, which may limit the detection of more subtle syntenic signals. Alternative approaches, such as pangenome graph-based methods, could in principle complement our analysis by capturing genome-wide structural variation and may help to further contextualize gene-level functional predictions. Nevertheless, our analyses provide a comprehensive framework for generating testable hypotheses and reveal previously unrecognized genetic features that may contribute to the ecological success of the SAR11 clade.

Looking forward, further advances in automated gene function annotation will be essential to keep pace with the rapid expansion of microbial genomic data and to complement meticulous but low-throughput manual annotation. By developing robust analytical approaches and moving beyond model organisms, we can uncover novel biological and ecological functions of microbes, providing deeper insight into the diversity and ecological roles of microbial life in natural environments. Bioinformatics-driven functional unknomics is poised to become a major frontier in microbial ecology, particularly for lineages that remain difficult to culture.

## Data Availability

The code and data sets generated and analyzed in this study are available in our Zenodo repository, https://doi.org/10.5281/zenodo.20669551.

## SUPPLEMENTAL MATERIAL

Supplemental figures msystems.01026-26-s0001.pdf Figures S1–S12; captions for Tables S1–S7 and for Supplemental text.

Supplemental text msystems.01026-26-s0002.docx Annotation results for the SAR11 conserved 108 unknome.

Supplemental tables msystems.01026-26-s0003.xlsx Tables S1 to S7.

ASM does not own the copyrights to Supplemental Material that may be linked to, or accessed through, an article. The authors have granted ASM a non-exclusive, world-wide license to publish the Supplemental Material files. Please contact the corresponding author directly for reuse.
