# Supplementary material for: Functional unknomics of the SAR11 clade reveal hidden genetic potential underlying adaptation to bottom-up and top-down pressures
Source: mSystems. 2026 Sep 17;11(10):e01026-26. doi: 10.1128/msystems.01026-26 (PMC13712998; doi:10.1128/msystems.01026-26)
Supplement: Supplementary file 1 [file msystems.01026-26-s0001.pdf]

Title

Functional unknomics of the SAR11 clade reveals hidden genetic potential underlying adaptation to bottom-up and top-down pressures

Authors

Satoshi Nishino[1,2], Kento Tominaga[3*], Kimiho Omae[4], Teppei Deguchi[5], Koji Hamasaki[1,2,6], Susumu Yoshizawa[2,3,6] and Yuki Nishimura[1*]

Affiliations

1) Department of Integrated Biosciences, Graduate School of Frontier Sciences, The University of Tokyo, 5-1-5 Kashiwanoha, Kashiwa, Chiba, 277-8562, Japan
2) Atmosphere and Ocean Research Institute, The University of Tokyo, 5-1-5 Kashiwanoha, Kashiwa, Chiba, 277-8564, Japan
3) Department of Natural Environmental Studies, Graduate School of Frontier Sciences, The University of Tokyo, 5-1-5 Kashiwanoha, Kashiwa, Chiba, 277-8563, Japan
4) RIKEN Pioneering Research Institute, 2-1 Hirosawa, Wako, Saitama, 351-0198, Japan
5) Department of Life Science, College of Science, Rikkyo University, 3-34-1 Nishi Ikebukuro, Toshima-ku, Tokyo, 171-8501, Japan
6) Collaborative Research Institute for Innovative Microbiology, The University of Tokyo, 1-1-1 Yayoi, Bunkyo-ku, Tokyo, 113-8657, Japan

Keywords

SAR11, Pelagibacterales, unknome, functionally unknown genes, antiviral defense system

*Correspondence should be addressed to K.T. (tominaga-kento@edu.k.u-tokyo.ac.jp) and Y.N. (ynishimura@edu.k.u-tokyo.ac.jp).

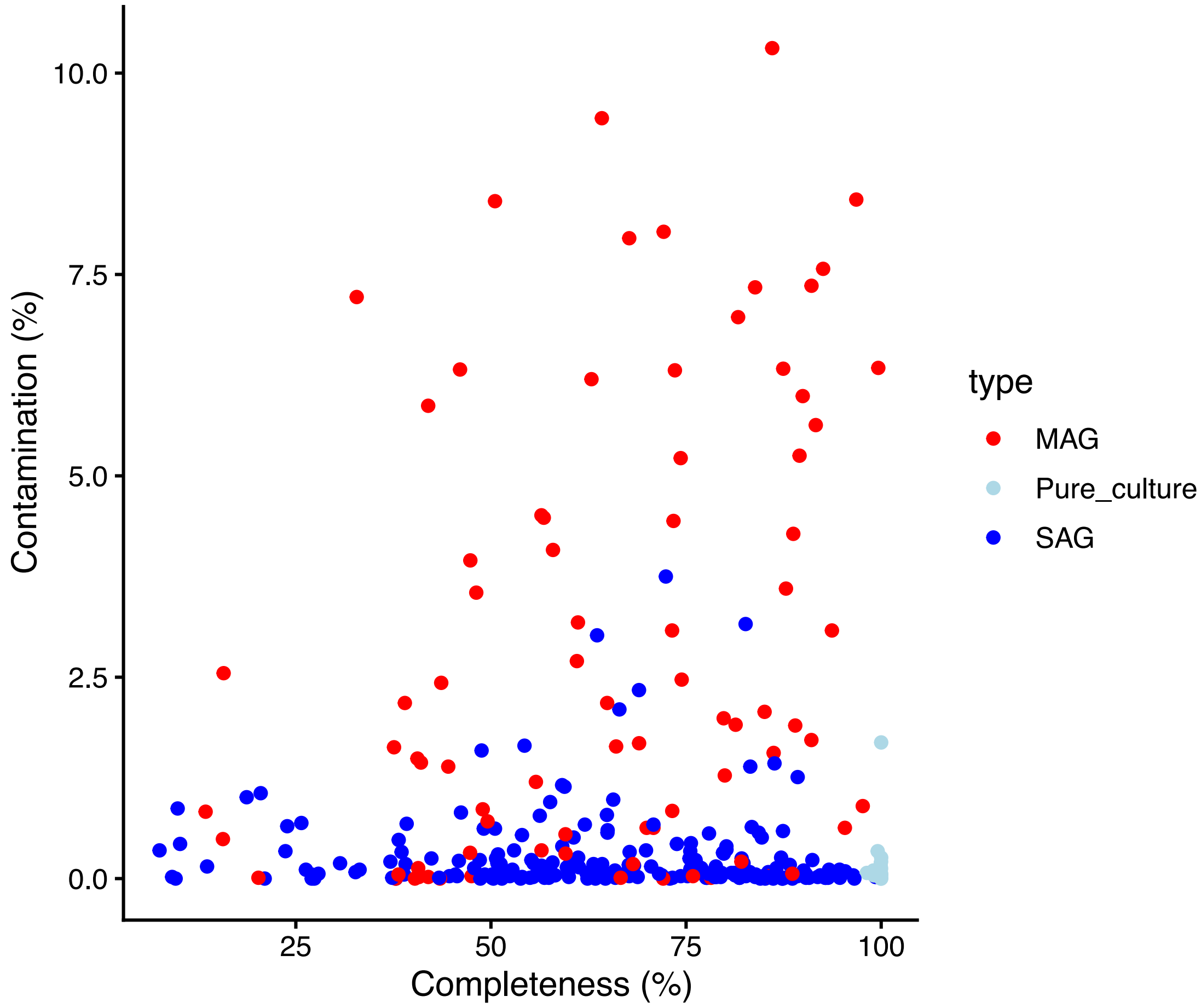

Supplemental Figure 1. Quality of the 321 genomes used in this study.
Scatter plot of the Completeness and Contamination scores calculated by CheckM2. The colors indicate genome types as follows: Metagenome-assembled genomes (red), Cultured strain (light blue), and Single-amplified genomes (blue).

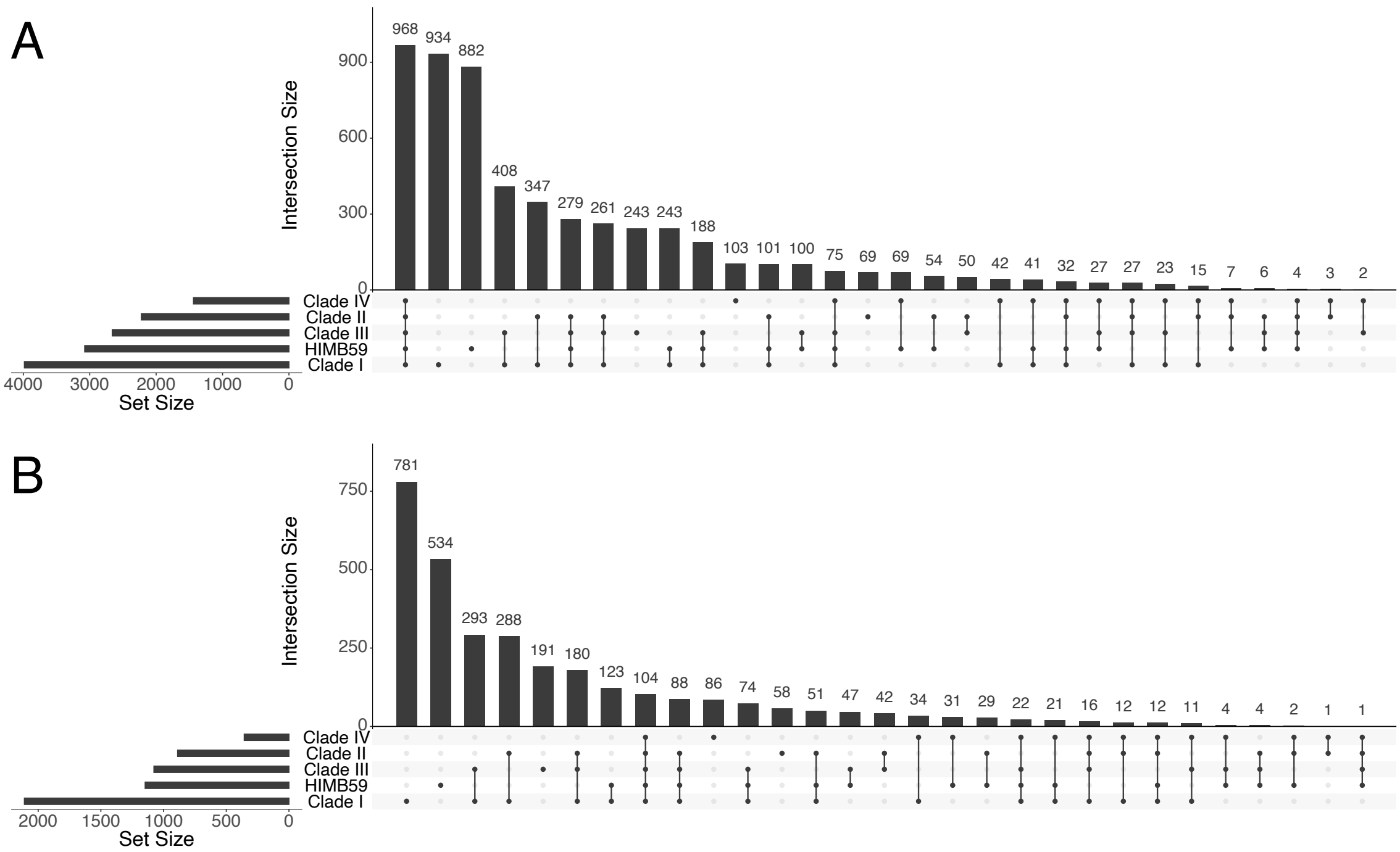

Supplemental Figure 2. Distribution of OGs among SAR11 and related clades
(A) Upset plot of all OGs distribution among the five clades. **(B)** Upset plot of the unknome OGs distribution among the five clades.

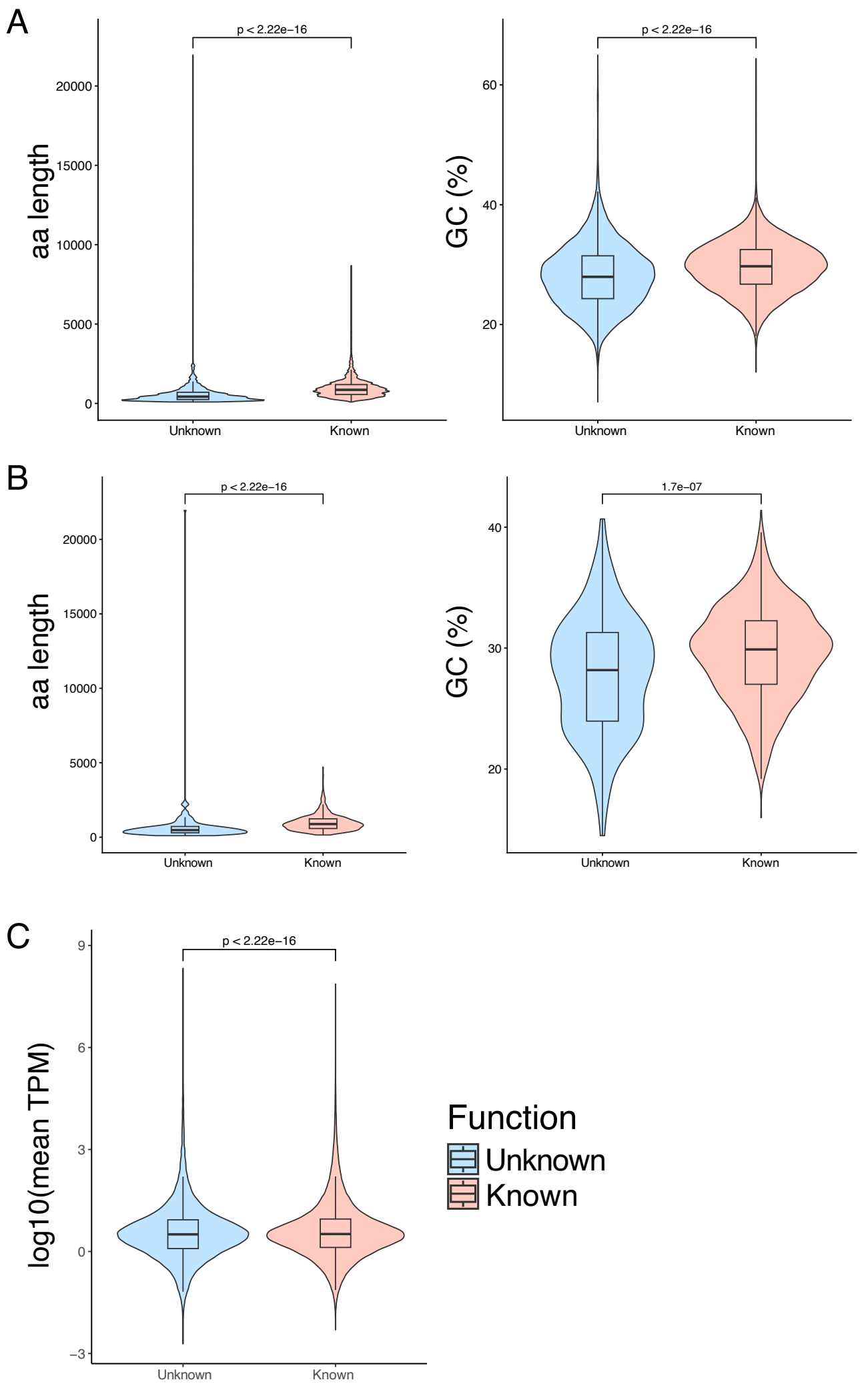

Supplemental Figure 3. Comparison of size and GC content between functionally known and unknown genes.

(A) The amino acid length and GC content distribution of all SAR11 genes analyzed in this study. **(B)** The amino acid length and GC content distribution of *Ca.* P. ubiqueversans HTCC1062 strain. **(C)** The average transcript per million (TPM) value of each gene calculated from the read mapping of Tara Oceans metatranscriptomic data to the SAR11 genomes analyzed in this study.

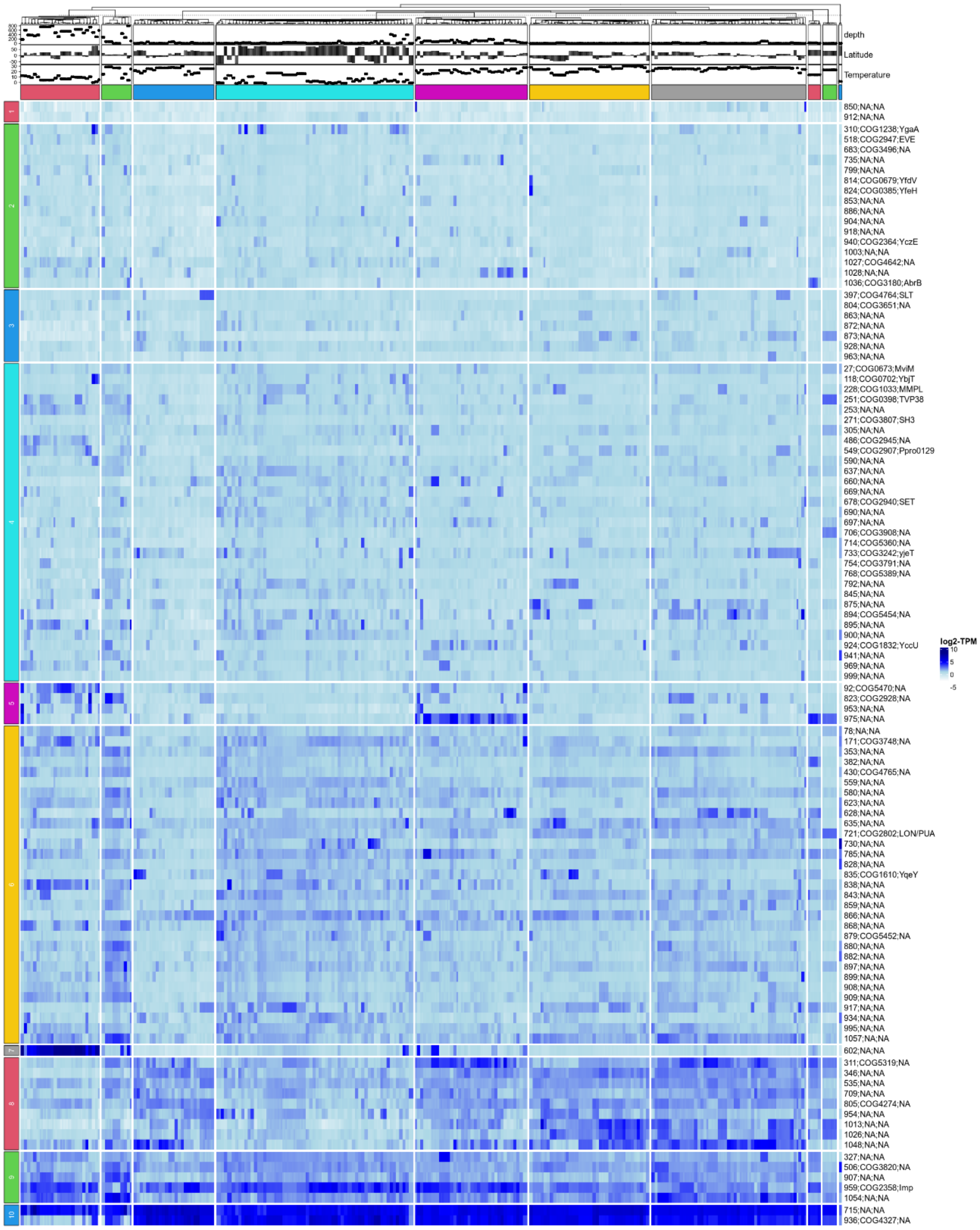

Supplemental Figure 4. Metatranscriptome expression profiles of the 108 conserved unknome OGs.

The heatmap shows expression profiles of each conserved unknome OGs based on TPM values. The heatmap was clustered using the default settings of ComplexHeatmap in R (n = 10, Euclidean distance and complete linkage). Plots above the heatmap indicate the

depth, latitude, and temperature for each sampling site. The vertical axis represents each conserved unknome OG and assigned COG information.

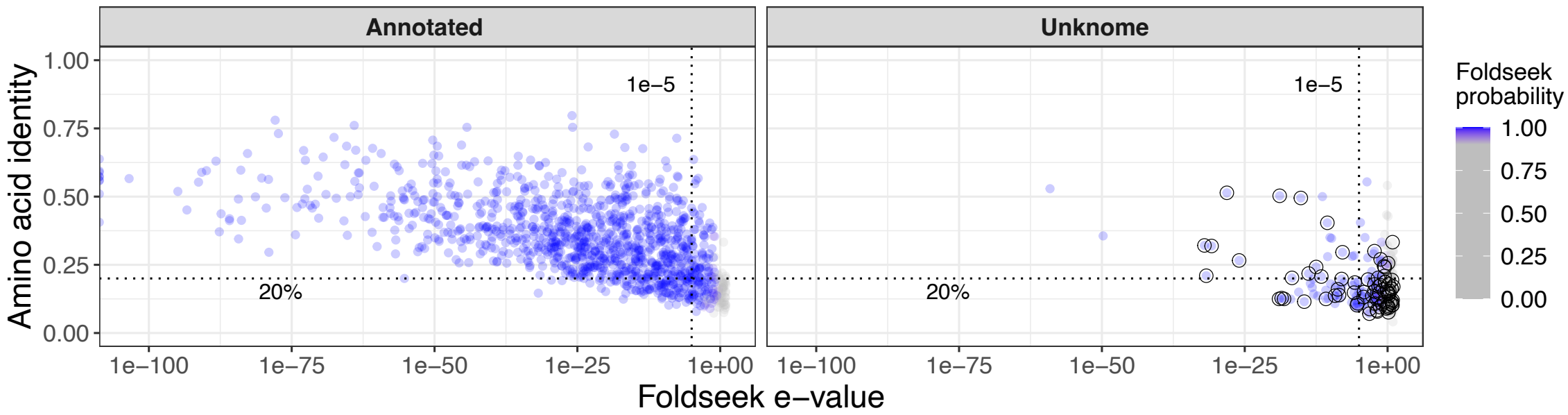

Supplemental Figure 5. Results of protein similarity search in IMCC9063 strain. Scatter plots of all IMCC9063 strain proteins compared with known protein structures in the Protein Data Bank. Dot colors indicate the Foldseek probability, and conserved unknome proteins are highlighted with outer black circles. Dotted lines represent thresholds of e-value = 1e−5 and amino acid identity = 20%, indicating potential remote homologs.

A

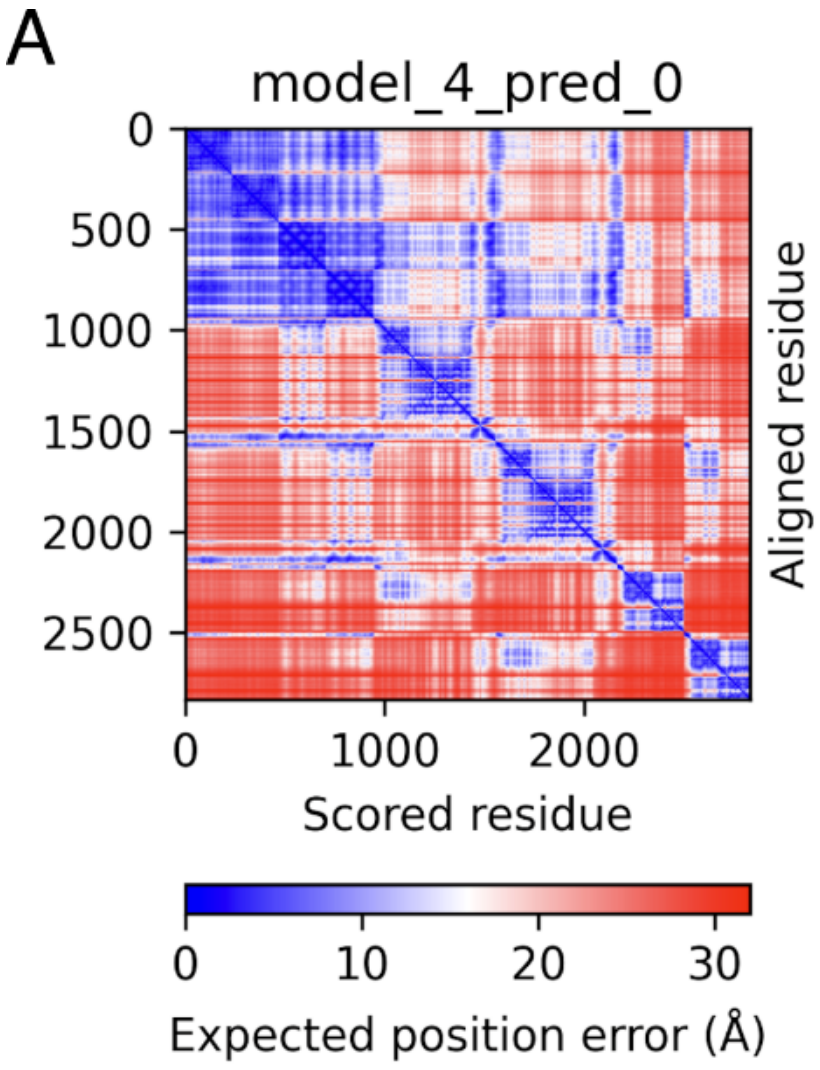

B

Chain	color	pDockQ_i
A (OG688)	pink	0.498444
B (OG688)	pink	0.509799
C (OG1772)	orange	0.529385
D (OG1772)	orange	0.523811
E (OG1815)	yellow	0.268428
F (OG1815)	yellow	0.274739
G (OG1814)	gray	0.363983
H (OG1814)	gray	0.336381

Supplemental Figure 6. Predicted aligned error plot and pDockQ2 scores of the putative transporter complex in Figure 2C.
(A) A predicted aligned error plot of the top-ranked predicted structure model. **(B)** pDockQ2 interface score of each chain. The minimum pDockQ2 interface score is 0.268 (chain E), indicating the potential for complex formation.

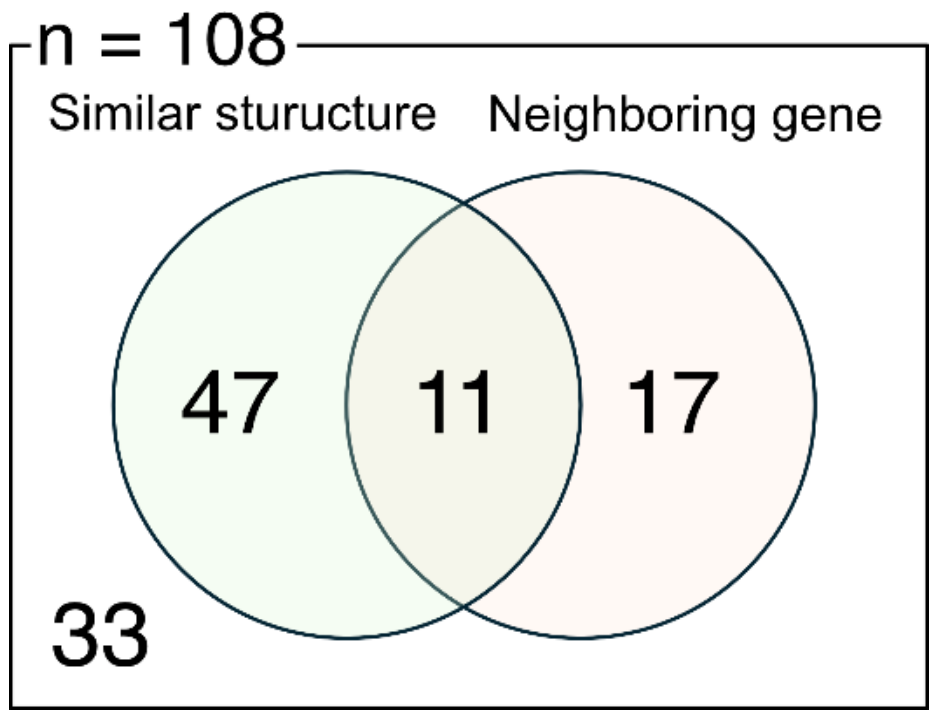

Supplemental Figure 7. Overlap of functional annotations obtained for the conserved unknome OGs by two approaches.
A Venn diagram shows the annotation results for 108 conserved unknome OGs in the SAR11 clade. Each circle represents the number of OGs that received a hit from a specific annotation approach: predicted structure similarity search to PDB (green) or neighborhood gene analysis (orange). Detailed functional investigations of each OG are provided in the Supplemental Text.

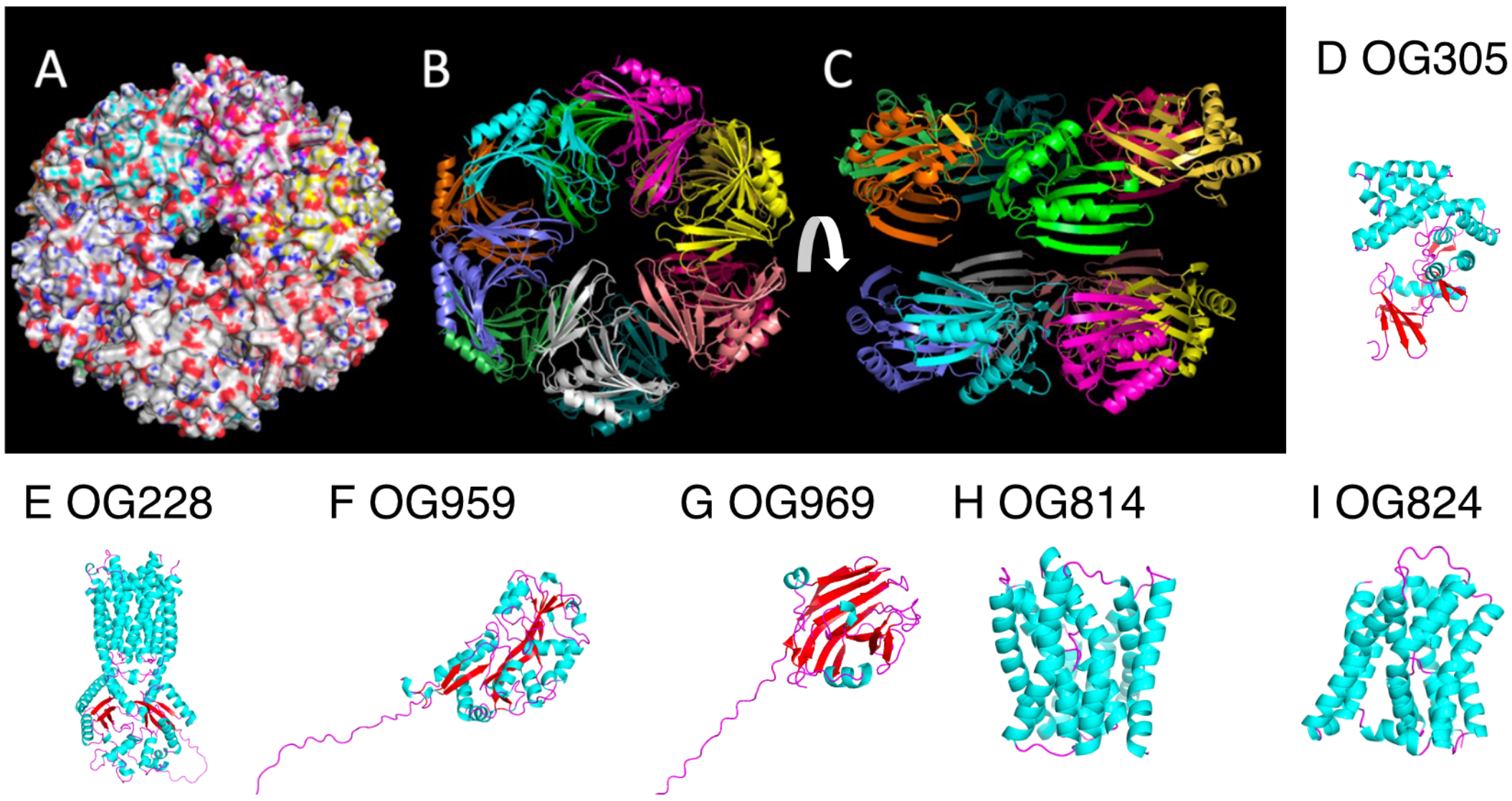

Supplemental Figure 8. Predicted unknome protein structures of putative transporter, channel, or metabolic enzyme.

(A-C) Predicted homo-12mer structure of OG305, putative channel protein, from *Ca.* P. ubiqueversans HTCC1062 strain. Each color shows each chain. (D-I) Predicted monomer structures of the unknomes from *Ca.* P. ubiqueversans HTCC1062 strain. Colors indicate secondary structure elements: alpha-helices (light blue), beta-sheets (red), and loops (pink). (D) OG305 monomer. (E) Putative AcrB-like transporter. (F) Putative substrate binding protein of TRAP-type transporter. (G) Putative glycan-processing enzymes. (H) Putative sodium/solute symporter. (I) Putative bile acid/sodium symporter.

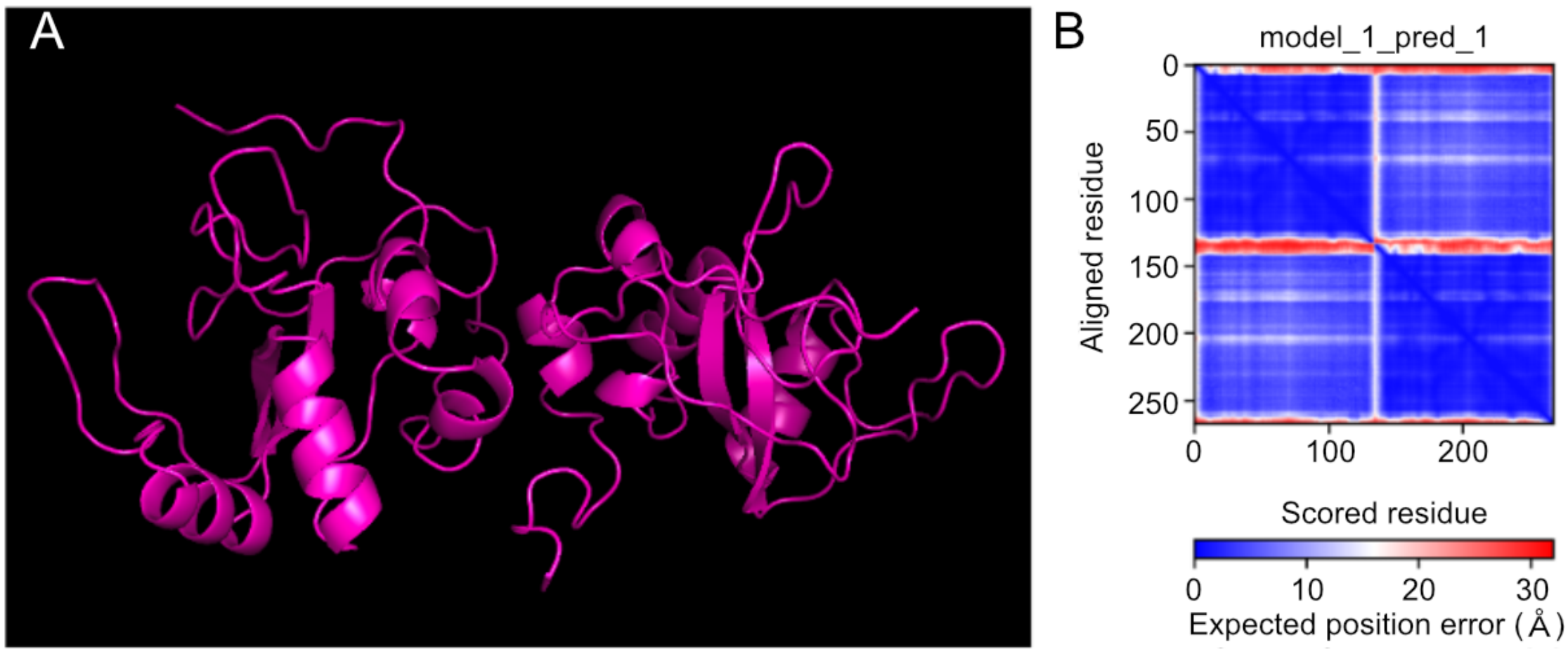

Supplemental Figure 9. Predicted DUF2237 homodimer structure.
(A) Predicted homo-dimer structure of DUF2237 protein on *Ca.* P. ubiqueversans HTCC1062 strain by AlphaFold2 multimer. **(B)** A predicted aligned error plot of the predicted homo-dimer structure of the DUF2237 protein.

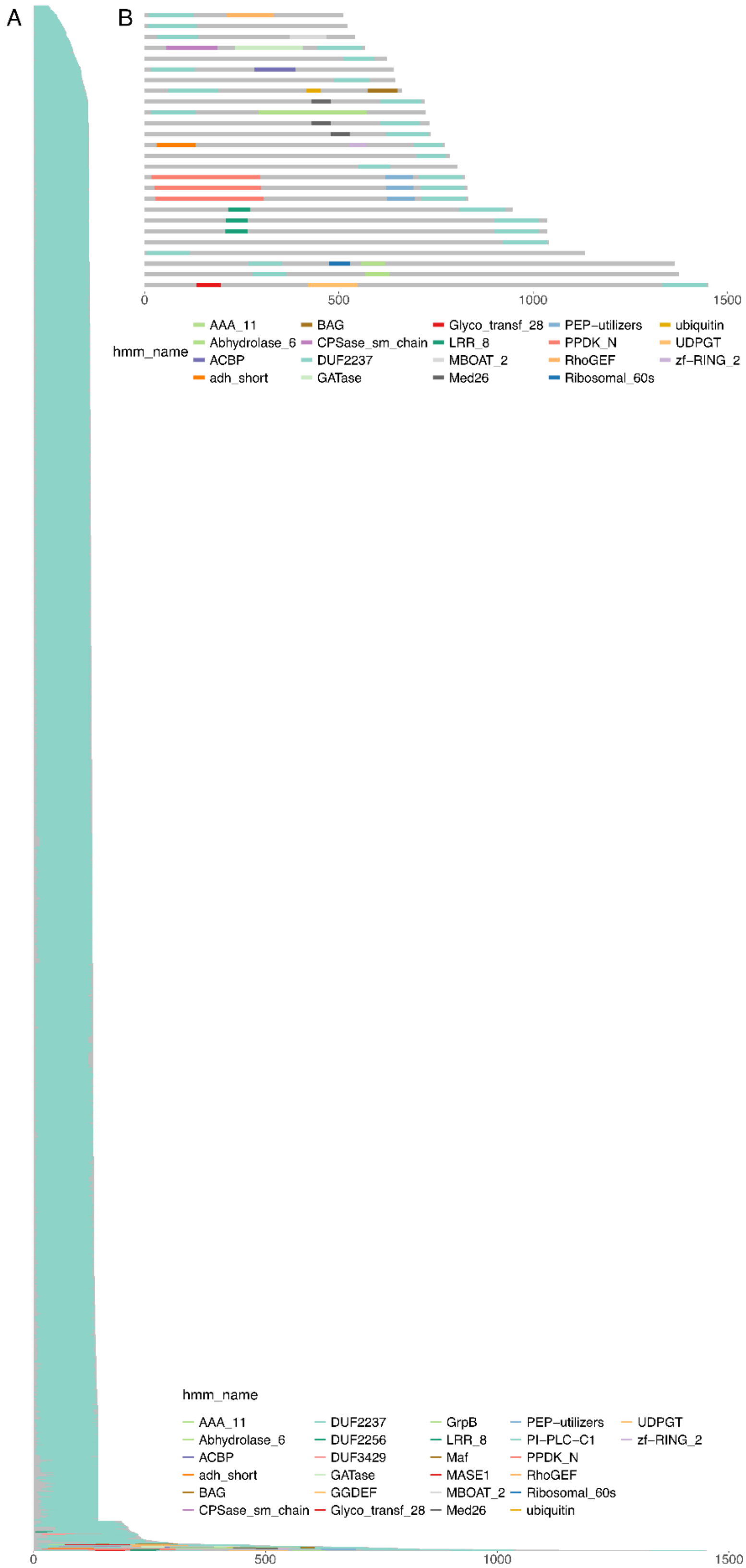
A
B
0
500
1000
1500
hmm_name
AAA_11
Abhydrolase_6
ACBP
adh_short
BAG
CPSase_sm_chain
DUF2237
GATase
Glyco_transf_28
LRR_8
MBOAT_2
Med26
PEP-utilizers
PPDK_N
RhoGEF
Ribosomal_60s
ubiquitin
UDPGT
zf-RING_2
hmm_name
AAA_11
Abhydrolase_6
ACBP
adh_short
BAG
CPSase_sm_chain
DUF2237
DUF2256
DUF3429
GATase
GGDEF
Glyco_transf_28
GrpB
LRR_8
Maf
MASE1
MBOAT_2
Med26
PEP-utilizers
PI-PLC-C1
PPDK_N
RhoGEF
Ribosomal_60s
ubiquitin
UDPGT
zf-RING_2
0
500
1000
1500

Supplemental Figure 10. Domain architecture of 7,968 DUF2237-containing proteins in the Pfam database.

(A) Domain architecture of 7,968 DUF2237-containing amino acid sequences in the Pfam database. The x-axis represents the length of the amino acid sequences, while the y-axis arranges the sequences in order of size. The colors indicate the presence of protein domains within each sequence. **(B)** Domain compositions of over 500 aa DUF2237-containing proteins in panel A.

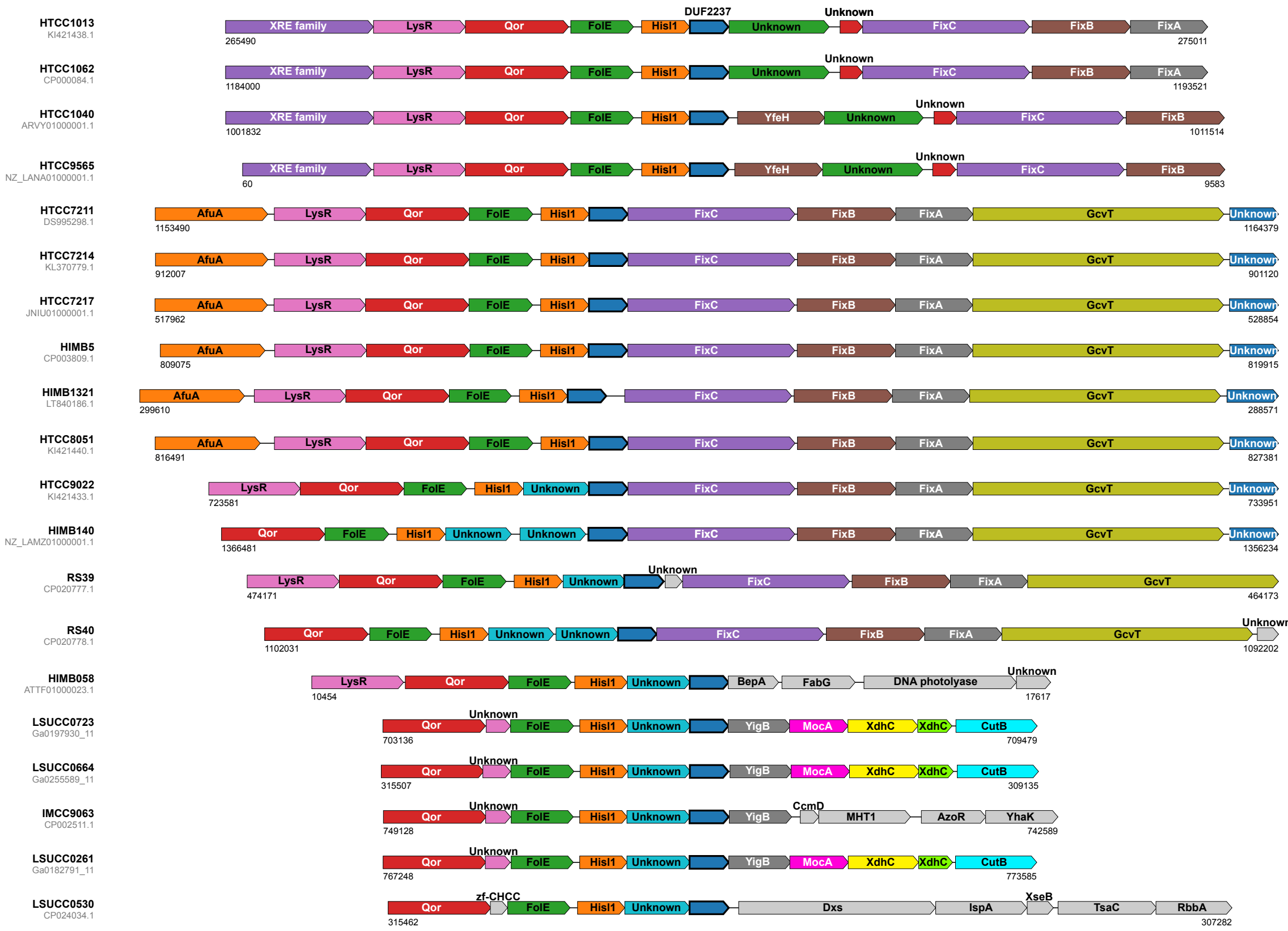

Supplemental Figure 11. Genomic context of DUF2237-containing genes in cultured SAR11 genomes.

Genomic regions encompassing the DUF2237-containing gene and the five upstream and downstream genes are shown for cultured SAR11 genomes. Genome and contig names are indicated for each region, together with the corresponding start and end coordinates. Each arrow represents a gene, and arrows sharing the same color correspond to the same OG. Light gray arrows indicate OGs that occur only once within the figure. The names and functional annotations of selected OGs shown in the figure are as follows: DUF2237, DUF2237-containing protein; HisI1, phosphoribosyl-AMP cyclohydrolase; FolE, GTP cyclohydrolase I; Qor, acrylyl-CoA reductase (NADPH); LysR, DNA-binding transcriptional regulator, LysR family; XRE family, XRE family transcriptional regulator; YfeH, predicted Na+-dependent transporter YfeH; FixC, electron-transferring-flavoprotein dehydrogenase; FixB, electron transfer flavoprotein alpha subunit; FixA, electron transfer flavoprotein beta subunit; GcvT, glycine cleavage system protein T (aminomethyltransferase); BepA, periplasmic chaperone/metalloprotease BepA/YfgC, contains M48 and TPR domains; FabG, NAD(P)-dependent dehydrogenase, short-chain alcohol dehydrogenase family; DNA photolyase, (6-4)DNA photolyase; YigB, FMN and 5-amino-6-(5-phospho-D-ribitylamino)uracil phosphatase YigB, HAD superfamily (riboflavin biosynthesis);

MocA, molybdenum cofactor cytidylyltransferase; XdhC, Molybdoenzyme maturation factor PaoD (Mo cofactor insertion), XdhC/CoxF family; CutB, Aldehyde, CO, or xanthine dehydrogenase, FAD-binding subunit; CcmD, heme exporter protein D; MHT1, homocysteine/selenocysteine methylase (S-methylmethionine-dependent); AzoR, FMN-dependent NADH-azoreductase; YhaK, quercetin 2,3-dioxygenase; Dxs, 1-deoxy-D-xylulose-5-phosphate synthase; IspA, Geranylgeranyl pyrophosphate synthase; XseB, exodeoxyribonuclease VII small subunit; TsaC, tRNA A37 threonylcarbamoyladenosine synthetase subunit TsaC/SUA5/YrdC; RbbC, Ribosome-associated ATPase or ATPase component of an ABC-type multidrug transport system.

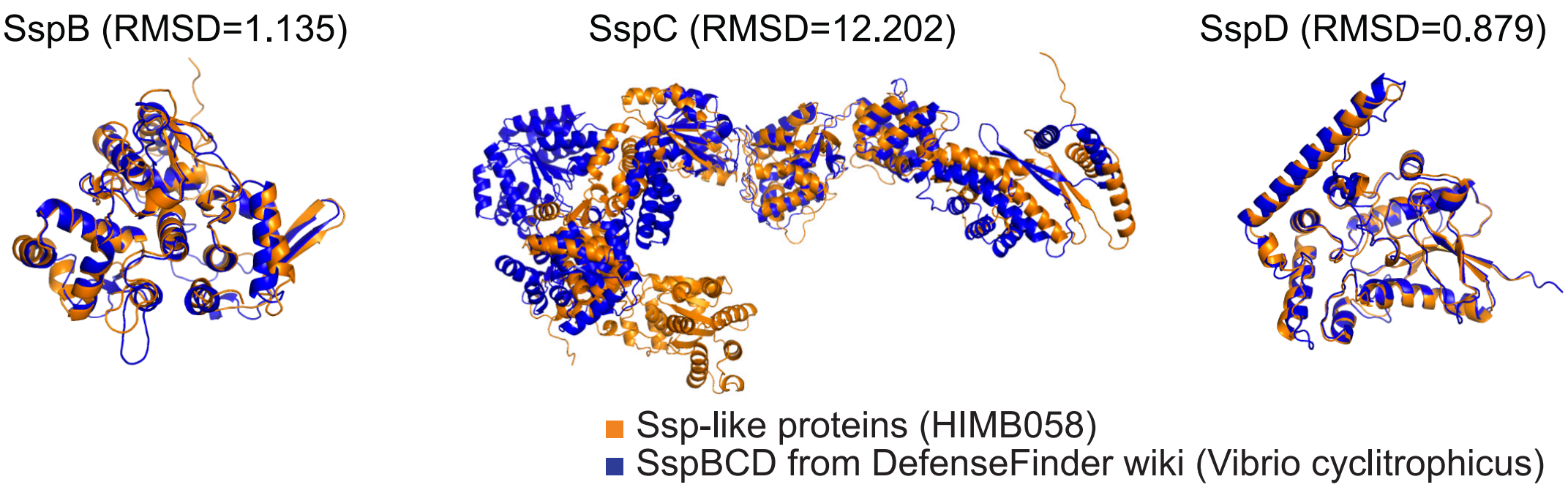

Supplemental Figure 12. Predicted structures of the SAR11 Ssp-like proteins
Predicted structure comparison of the known SspBCD proteins from DefenseFinder wiki (blue) and the Ssp-like proteins in HIMB058 (orange).

Supplemental Tables

Table S1. SAR11 genomes used in this study.

Table S2. List of ENA_Run_IDs used for the metatranscriptomic read mapping.

Table S3. Docking simulation results and compounds list.

Table S4. Sequence similarity-based ortholog annotation results.

Table S5. Function prediction summary of the 108 conserved unknome OGs.

Table S6. Raw data of neighboring gene analysis result.

Table S7. SAR11 defense systems found in this study.

Supplemental Text

Annotation results for the 108 conserved unknome OGs.
