# Supplementary material for: Functional unknomics of the SAR11 clade reveal hidden genetic potential underlying adaptation to bottom-up and top-down pressures
Source: mSystems. 2026 Sep 17;11(10):e01026-26. doi: 10.1128/msystems.01026-26 (PMC13712998; doi:10.1128/msystems.01026-26)
Supplement: Supplementary file 2 [file msystems.01026-26-s0002.docx]

# **Supplemental manuscript for “Functional unknomics of the SAR11 clade reveals hidden genetic potential underlying adaptation to bottom-up and top-down pressures”**

**Authors**

Satoshi Nishino^1,2^, Kento Tominaga^3*^, Kimiho Omae^4^, Teppei Deguchi^5^, Koji Hamasaki^1,2,6^, Susumu Yoshizawa^2,3,6^ and Yuki Nishimura^1*^

**Affiliations**

1) Department of Integrated Biosciences, Graduate School of Frontier Sciences, The University of Tokyo, 5-1-5 Kashiwanoha, Kashiwa, Chiba, 277-8562, Japan
2) Atmosphere and Ocean Research Institute, The University of Tokyo, 5-1-5 Kashiwanoha, Kashiwa, Chiba, 277-8564, Japan
3) Department of Natural Environmental Studies, Graduate School of Frontier Sciences, The University of Tokyo, 5-1-5 Kashiwanoha, Kashiwa, Chiba, 277-8563, Japan
4) RIKEN Pioneering Research Institute, 2-1 Hirosawa, Wako, Saitama, 351-0198, Japan
5) Department of Life Science, College of Science, Rikkyo University, 3-34-1 Nishi Ikebukuro, Toshima-ku, Tokyo, 171-8501, Japan
6) Collaborative Research Institute for Innovative Microbiology, The University of Tokyo, 1-1-1 Yayoi, Bunkyo-ku, Tokyo, 113-8657, Japan

# This manuscript contains annotation results for the SAR11 conserved 108 unknome.

# 1. **Functions could be estimated from combined structural similarity search, neighboring genes, and literature information (7 OGs)**

**OG27 MviM**

OG27 was annotated as COG0673 and identified as the gene known as mviM. There are four previous studies on MviM, all of which report its inclusion in sugar catabolic operons and its involvement in redox reactions targeting specific hydroxyl groups of sugars (1–4). However, types of sugars and the neighboring sugar metabolism genes vary among these studies. In the case of SAR11, the ligand could not be inferred from the genome context. Structural predictions revealed that MviM possesses a Rossmann fold, a nucleotide-binding domain, confirming that it is an NADH-dependent oxidoreductase (5).

**OG353 Tol-Pal system inner membrane component**

OG353 was initially classified as a functionally unknown membrane protein. Structural similarity analysis revealed that it shares a similar fold with TolA, a component of the Tol-Pal system (e.g., PDB entries 3QDR, 1S62). In its genomic neighborhood, an operon containing genes related to the Tol-Pal system, including tolB, tolQ, and ompA, was conserved. These findings suggest that OG353 is likely a remote homolog of TolA.

**OG549 Putative light-related oxidoreductase**

OG549 was annotated as COG2907, a gene of previously unknown function. However, structural predictions revealed that it is an NAD⁺/FAD⁺-binding oxidoreductase. In the gamma-proteobacterium *Acinetobacter baumannii*, transcription of the operon containing this gene has been shown to increase under blue light exposure (6). In Aeromonas salmonicida, another gammaproteobacterium, transcription of the gene decreases when cultured in riboflavin-supplemented medium, suggesting a link to fatty acid metabolism (7). A similar operon structure, including OG683, is also observed in SAR11. However, previous studies culturing SAR11 under light conditions did not detect any significant increase in transcription (8).

**OG683 Putative pigment synthesis gene**

OG683 was annotated as K09701 and COG3496, and contains a DUF1365 domain, classifying it as a domain of unknown function. In the gammaproteobacterium *A. baumannii*, transcription of this gene is known to increase under blue light exposure (6). In a previous study on *A. salmonicida*, transcription levels decreased when cultured in riboflavin-supplemented medium, suggesting a potential role in fatty acid metabolism (7). Additionally, a possible association with the carotenoid lutein has been suggested in Cucurbita (pumpkin) (9). In contrast, no significant increase in transcription was observed in SAR11 when cultured under light conditions (8). Structural similarity analysis revealed that OG683 shares a similar fold with the fatty acid-binding protein Ycf58 (PDB 3BDR). Taken together, these findings suggest that OG683 may be involved in pigment biosynthesis related to light utilization.

**OG866 Membrane anchor protein AprM**

OG866 was initially classified as a functionally unknown membrane protein. However, the presence of neighboring genes encoding adenylylsulfate reductase subunit beta (aprB) and subunit alpha (aprA) suggested that OG866 may be aprM—the membrane anchor of the adenosine-5′-phosphosulfate reductase (AprMAB) complex, which catalyzes the oxidation of sulfite to sulfate (10). To test this hypothesis, we performed a complex structure prediction using the amino acid sequences of these three genes by AlphaFold-multimer, which successfully yielded a predicted complex. No known proteins with a similar structure were found in the PDB. This complex is known to catalyze the oxidation of sulfite to sulfate (11).

**OG882 Exonuclease VII small subunit protein**

Sixteen out of 173 sequences showed sequence similarity to the Exonuclease VII small subunit in the COG database (maximum identity: 34.2%), and one sequence was annotated as COG4942 (EnvC; identity: 29.4%). The predicted compact hairpin-like helical structure of OG882 is also consistent with that of the Exonuclease VII small subunit, supporting the possibility that OG882 represents a homolog of this protein. This hypothesis is further supported by annotations in the STRING database (e.g., AAZ21430.1), where some of these sequences are also predicted to encode Exonuclease VII small subunits.

**OG909 Cell division protein FtsL**

In SAR11 genomes, the neighboring gene OG169 (COG0769; UDP-N-acetylmuramoyl tripeptide synthase) is conserved, suggesting a potential link to cell envelope biosynthesis. Among the 163 sequences classified under OG909, four showed significant similarity to COG4839 (maximum amino acid identity: 31.3%), suggesting that OG909 may be a homolog of FtsL2, a cell division protein FtsL. However, similarities were also observed with other COGs, including COG4942 (30.3% identity), COG5462 (31.8% identity), and COG1593 (21.3% identity), making it difficult to predict the function of OG909 based solely on database annotation. A structural similarity search using the predicted 3D structure of OG909 from the *Ca.* P. ubique HTCC1062 strain revealed structural similarity to the well-characterized *E. coli* FtsL protein (12) (Foldseek probability = 0.98), although the amino acid sequence identity remained low at 18.8% (see figure below). These results suggest that OG909 may represent a remote homolog of FtsL. These results were further supported by neighboring gene information from the STRING database.

# 2. **Functions are known or predictable from literature information (7 OGs)**

**OG310 DedA family flippase PetA（YgaA）**

OG310 was annotated as COG1238 and identified as a membrane protein of the DedA superfamily, which is commonly found in Gram-negative bacteria. In March 2023, it was suggested that this protein interacts with the phospholipid-bridging protein AsmA to mediate phospholipid flipping (13). Subsequently, in May 2023, its function as a phosphatidylethanolamine transporter was experimentally confirmed in *Bacillus subtilis*, and the protein was named PetA (14).

**OG506 Transcriptional regulators including DUF1013, TrcR**

OG506 encodes a protein containing the DUF1013 domain, a domain of unknown function. In SAR11 genomes, two neighboring genes—OG507 and OG442—encode the non-essential large subunit ribosomal protein L31 and the Elongation Factor P, respectively, suggesting that the functionally uncharacterized OG506 may be related to translation. A previous study screening cell cycle mutants of the model alpha-proteobacterium *Caulobacter crescentus* identified a DUF1013-containing protein that binds RNA polymerase and affects both transcription initiation and elongation, and this protein was named TrcR (15). In bacteria, it is well established that transcription elongation by RNA polymerase and translation by ribosomes are spatially and temporally coupled (16,17), raising the possibility that OG506 may be involved in this mechanism.

**OG518 RNA binding protein**

OG518 was annotated as COG2947 and identified as a protein of unknown function containing an EVE domain. Structural similarity analysis revealed that it shares structural features with RNA-binding proteins, such as the one represented by PDB entry 2GBS. The EVE domain is known to function as an RNA-binding motif (18). Two evolutionary types of EVE domains have been described—one evolving rapidly and the other more slowly—and the latter is found in alpha-proteobacteria, including the SAR11 clade (19). Proteins containing the alphaproteobacterial-type EVE domain are suggested to be involved in translation, respiration, and cytochrome c binding through recognition of 5-methylcytosine modifications in tRNA (19).

**OG637 Phospholipid bridge to outer membrane**

OG637 was identified as a gene of unknown function containing the DUF3971 domain. Recent studies have suggested that its *E. coli* homolog, YhdP, is involved in maintaining outer membrane phospholipid homeostasis (20,21). In October 2023, a preprint was published that elucidated its molecular mechanism, reporting that YhdP functions as a phospholipid bridge to the outer membrane (22).

**OG785 BamC homolog of Alphaproteobacteria, BamF (DUF3035)**

OG785 encodes a protein containing a DUF3035 domain, and its genomic neighborhood includes OG490 (COG0742; 16S rRNA G966 N2-methylase RsmD). DUF3035 is an uncharacterized domain found exclusively in Alphaproteobacteria and is known as BamF. BamF is a lipoprotein component of the outer membrane β-barrel assembly machinery (BAM) complex (23).

**OG845 DCC family thioredoxin**

OG845 was identified as a gene of unknown function containing the DUF393 domain. DUF393 is known to share structural similarity with thiol-disulfide oxidoreductases (24), suggesting that OG845 also functions as a thioredoxin.

**OG940 Possibly involved in the transport of sulfur-containing compounds**

OG940 is annotated as COG2364 and K07149 and is homologous to the uncharacterized Escherichia coli protein *yczE*. Comparative genomic analysis of *yczE* has previously been conducted to predict its function (25). Because genes that co-occur with *yczE* are associated with sulfur metabolism, *yczE* has been proposed to be involved in the membrane translocation and metabolism of sulfur-containing compounds. Therefore, OG940 is also predicted to participate in these processes.

# 3. **Function predicted by predicted structure (43 OGs)**

**OG78 Methyltransferase**

OG78 is an uncharacterized gene containing a methyltransferase domain. Structural similarity analysis revealed resemblance to multiple methyltransferase proteins, including tRNA methyltransferase (PDB: 8K1F).

**OG92 DUF1330-containing cofactor-independent oxygenase**

OG92 harbors a domain of unknown function, DUF1330. DUF1330 is primarily found in Proteobacteria and Actinobacteria and has been shown to be essential for the biosynthesis of L-allo-isoleucine, a precursor of coronatine, in Pseudomonas (26). A structural similarity search revealed three experimentally determined structures (PDB: 2FIU, 3LO3, 3DCA), all of which remain functionally unannotated. These structures are all deposited as homodimers. Notably, the structural features resemble those of cofactor-independent monooxygenases such as SnoaB and LsrG (27), suggesting that OG92 may function as an oxygenase capable of utilizing molecular oxygen directly.

**OG118 DUF2867-containing redox enzyme associated with the NADH:ubiquinone oxidoreductase complex**

OG118 is an uncharacterized gene also known in *Escherichia coli* as YbjT (COG0702). This protein harbors the DUF2867 domain. Structural prediction revealed the presence of a nucleotide-binding motif consistent with a Rossmann fold (5), and no transmembrane helices were predicted. A structural similarity search indicated that OG118 shares significant structural homology with the NADH-binding proteins of the eukaryotic respiratory complex I. Furthermore, co-expression data from the STRING database suggest a potential association with bacterial complex I gene clusters (NuoABCDGIM). Collectively, these findings suggest that OG118 may function as a redox enzyme associated with the NADH:ubiquinone oxidoreductase complex.

**OG253 Fe(II)/2-oxoglutarate-dependent dioxygenase**

OG253 is a protein possessing an Fe2+/2-oxoglutarate (2OG)-binding domain. Structural similarity analysis revealed homology to multiple Fe2+/2OG-binding proteins (e.g., PDB ID: 2RG4).

**OG271 Dipeptidyl peptidase**

OG271 is an uncharacterized gene annotated as COG3807. The encoded protein consists of a signal peptide and two SH3 domains, which are known to interact with proline-rich sequences (28). Structural comparison revealed similarity to putative dipeptidyl peptidase IV or VI enzymes derived from Bacteroides species (PDB: 4R0K, 3NPF, 3PVQ). Dipeptidyl peptidase IV is an N-terminal protein-processing enzyme that specifically cleaves dipeptides from the N-terminus when the second residue is a proline or alanine (29).

**OG228 AcrB-like transporter**

OG228 is an uncharacterized transporter gene containing two MmpL domains, which are known membrane transport domains (30). On the SAR11 genome, the DNA helicase gene dnaB is located in close proximity. Structural similarity analysis revealed a high degree of homology with transporters containing MmpL3 domains (e.g., PDB: 7N6B). OG228 also shows partial sequence and structural similarity to the multidrug efflux transporter AcrB. AcrB functions as an inner membrane pump by forming a complex with the adaptor protein AcrA and connects to outer membrane channels such as TolC or OprM to mediate multidrug efflux (31). However, neither acrA nor tolC homologs are found near OG228, and another copy of acrB is located adjacent to acrA in the SAR11 genome. These observations suggest that OG228 may transport substrates distinct from those of AcrB.

**OG346 Hydroxylase**

Structural similarity analysis revealed that OG346 shares structural homology with multiple hydroxylases (e.g., PDB: 7V4O, 4P7W), suggesting that it functions as a hydroxylase.

**OG397 Lytic transglycosylase**

OG397 is an uncharacterized gene annotated as COG4764 and encodes a protein with a predicted signal peptide. Structural similarity analysis revealed homology with several lytic transglycosylases (e.g., PDB: 5O1J, 5OHU, 5O29). Lytic transglycosylases are enzymes that degrade peptidoglycan by cleaving free glycan chains, thereby preventing their accumulation in the periplasmic space (32). These enzymes are also known to create localized gaps in the peptidoglycan layer to allow insertion of structures such as the flagellar basal body (33). COG4764 sequences have been detected in marine environments, suggesting that it represents a gene specific to marine microorganisms.

**OG430 DUF2155-containing tyrosine phosphatase**

OG430 is a gene annotated as COG4765 and contains the domain of unknown function DUF2155. A previous study that clustered marine bacterial genes based on abundance correlations across sampling sites suggested that DUF2155 belongs to a cluster of genes involved in catalyzing the cleavage of nitrogen-containing moieties from polyamines and purines (34). On the SAR11 genome, the NADH-ubiquinone oxidoreductase gene NDUFA12 was found close to OG430. Structural similarity analysis revealed that OG430 shares significant structural homology with several protein tyrosine phosphatases (e.g., PDB: 4GE5, 6KRX), suggesting that OG430 may function as a tyrosine phosphatase.

**OG486 α/β hydrolase**

OG486 is an uncharacterized gene annotated as K07018 and COG2945. Structural similarity searches yielded hits to approximately 900 protein structures, including PDB 2I3D, with many of the top-ranked structures belonging to the α/β hydrolase superfamily. The α/β hydrolase superfamily is characterized by a conserved fold consisting of an eight-stranded β-sheet flanked by several α-helices, but despite this common fold, its members exhibit diverse evolutionary origins and functions (35).

**OG559 Putative ribonucleotide reductase**

The predicted structure of OG559 protein showed similarity to multiple vitamin B12-dependent ribonucleotide reductases registered in the AlphaFold DB (36), suggesting that it may function as a ribonucleotide reductase.

**OG580 Cell cycle regulator**

OG580 is an uncharacterized gene containing a zinc ribbon motif. Structural similarity analysis revealed that the C-terminal zinc ribbon domain is homologous to that of ZitP (PDB: 2NB9) from *C. crescentus*. ZitP is known to be involved in pilus biogenesis and motility, and it has been shown to regulate cell polarity through interaction with PopZ (37–39).

**OG660 Lipoprotein**

OG660 is an uncharacterized gene encoding a protein with a predicted signal peptide. Structural similarity analysis revealed homology with a lipid-binding protein (PDB: 3BDR), suggesting that OG660 may function as a lipoprotein.

**OG628 Prolyl hydroxylase**

OG628 is an uncharacterized gene with structural similarity to proteins containing a 2-oxoglutarate (2OG)- and Fe²⁺-binding domain. Structural similarity analysis revealed homology with the eukaryotic 2OG-dependent prolyl hydroxylase (PDB 4NHY) (40), and the prokaryotic elongation factor Tu prolyl hydroxylase (PDB 3IW3). These enzymes are believed to share a common evolutionary origin (41). OG628 is therefore predicted to be a prolyl hydroxylase of the same origin.

**OG635 DUF3576-containing lipoprotein**

OG635 is an uncharacterized gene encoding a protein with a predicted signal peptide and the domain of unknown function DUF3576. Structural similarity analysis revealed homology with the outer membrane lipoprotein BamC (PDB 2YH6, 6LYS) (42).

**OG678 Methyltransferase**

OG678 is an uncharacterized gene annotated as COG2940. Structural similarity analysis revealed homology to multiple eukaryotic histone methyltransferases (e.g., PDB 7W67, 2W5Z). Recent studies have shown that some proteobacteria possess histone-like proteins (43); however, it remains unclear whether SAR11 harbors histone-like proteins.

**OG709 Lipoprotein**

OG709 is a gene of unknown function that contains a predicted signal peptide. Structural analysis revealed similarity to lipoproteins, such as the lipoprotein from *Pseudomonas aeruginosa* PAO1 (PDB 4DM5), suggesting that OG709 encodes a lipoprotein.

**OG714 HepC, heparinase II/III-like**

OG714 is an uncharacterized gene annotated as COG5360 (Uncharacterized conserved protein, heparinase superfamily). Notably, 75.8% of COG5360 homologs have been detected in marine environments. Structural similarity to the heparan sulfate lyase from *Pedobacter heparinus* (PDB 4MMI) suggests that OG714 may function as a HepC-like enzyme (44).

**OG715 OmpF1-like porin**

OG715 is a gene of unknown function lacking annotation in COG databases. In SAR11 genomes, OG715 is syntenically conserved with neighboring genes OG635 (encoding a DUF3576-containing lipoprotein) and OG701 (COG3786, L,D-peptidoglycan transpeptidase). Predicted structural similarity to the *E. coli* porin OmpF1 (PDB 7FDY) suggests that OG715 functions as an OmpF1-like porin.

**OG721 Lon protease**

OG721 is an uncharacterized gene annotated as K07157 and COG2802. Structural similarity analysis revealed that it shares structural homology with ATP-dependent serine protease Lon protease (PDB: 7CR9, 3LJC) (45).

**OG735 RuvC endodeoxyribonuclease**

OG735 is an uncharacterized gene without a corresponding annotation in the COG database. Predicted structural similarity to multiple endodeoxyribonucleases, including the protein represented by PDB 6JRF, indicates that OG735 may function as an endodeoxyribonuclease.

**OG768 Helicase loader DciA**

OG768 is a gene annotated as COG5389 and contains the DUF721 domain. Structural similarity analysis revealed homology to DciA (PDB 7YKM, 8A3V), a helicase loader protein that facilitates the loading of the DNA helicase onto the replication origin during replication initiation (46,47).

**OG814 Na+/solute symporter**

OG814 is a membrane protein gene of unknown function, annotated as COG0679 (YfdV). Structural predictions revealed similarity to known Na⁺/solute symporters, such as SbtA and a Na⁺/bile acid symporter (PDB 7CYF, 4N7W), suggesting that OG814 may function as a Na⁺/solute symporter.

**OG824 Bile acid sodium symporter homolog**

OG824 is an uncharacterized membrane protein gene annotated as COG0385 (YfeH). Its predicted structure shows similarity to the bile acid sodium symporter from *Neisseria meningitidis* (PDB 3ZUX), suggesting that OG824 may be a homolog of this transporter.

**OG828 SurA-like chaperone**

OG828 is an uncharacterized membrane protein. Structural similarity analysis revealed homology to SurA-like chaperones (e.g., PDB: 3RGC). SurA is a chaperone that facilitates the folding of outer membrane proteins (48). The genomic neighborhood of OG828 includes SecG, a subunit of the Sec translocase, and triosephosphate isomerase TpiA on the SAR11 genomes.

**OG838 Putative sulfotransferase**

OG838 contains a Sulfotransfer_1 domain (Pfam PF00685), and structural similarity searches suggested that it is a putative sulfotransferase (PDB 2REO).

**OG853 VipF acetyltransferase**

OG853 is an uncharacterized gene lacking functional annotation in the COG database. Predicted structural similarity to several acetyltransferases (e.g., PDB 6WQB) suggests that OG853 may encode an acetyltransferase.

**OG859 LptE lipoprotein**

OG859 is an uncharacterized gene containing a predicted signal peptide. In SAR11 genomes, it is consistently located near OG645 (COG0495, leucyl-tRNA synthetase). Structural similarity to LptE from Pseudomonas aeruginosa (PDB 8H1R) suggests that OG859 encodes a lipoprotein.

**OG868 Cell division-related protein FtsB**

OG868 is an uncharacterized membrane protein gene. In April 2023, the structure of FtsB, a protein component of the bacterial divisome complex with structural similarity to OG868, was released in the PDB (PDB ID: 8HHG) (12). In *E. coli*, FtsB is an essential gene known to form the FtsBLQ complex, which localizes FtsI and FtsW to the Z-ring (49).

**OG879 Chaperone**

OG879 is an uncharacterized gene annotated as COG5452. Structural similarity analysis revealed homology to CBP3, a ubiquinol–cytochrome c chaperone (PDB: 6RWT) (50).

**OG900 Succinate dehydrogenase protein subunit E (SdhE)**

Structural similarity analysis revealed that OG900 is homologous to *E. coli* ygfY (PDB 1X6J), a protein involved in FAD assembly (SdhE) (51).

**OG908 FtsQ-like SPOR domain-containing protein**

OG908 is an uncharacterized gene containing a predicted signal peptide region. Structural similarity searches revealed homology to proteins containing a SPOR domain (e.g., PDB: 4AVR, 6I05), suggesting that OG908 may be a homolog of FtsQ, a protein involved in bacterial cell division.

**OG917 HTH deoR-type domain-containing protein**

OG917 showed structural similarity to an HTH DeoR-type domain-containing protein (PDB 2O0Y), indicating its potential role as a transcription factor.

**OG924 Putative acetyl donor *yccU* homolog**

OG924 is annotated as COG1832 and K06929 and corresponds to the uncharacterized gene *yccU* in Escherichia coli, which has been described as a putative HspQ acetyl donor. Therefore, OG924 may also be involved in acetyl donor-related functions.

**OG953 Putative metal-dependent oxidoreductase (manganese-containing cupin)**

OG953 is structurally similar to TM1459, a manganese-containing cupin protein from *Thermotoga maritima* (PDB: 8AWN). TM1459 has been reported to catalyze the oxidative cleavage of styrene derivatives in vitro (52), suggesting a role as a metal-dependent oxidoreductase.

**OG959 Substrate binding protein of TRAP-type transporter**

OG959 is annotated as COG2358 and K07080. Structural similarity searches identified similarity to the taurine transporter substrate-binding protein TauA from *Escherichia coli* in complex with 2-(N-morpholino)ethanesulfonic acid (MES) (PDB 6ST1). Based on this structural similarity, OG959 is predicted to function as a substrate-binding component of a TRAP-type transporter.

**OG963 PD-(D/E)XK superfamily nuclease**

OG963 is structurally similar to the *Escherichia coli* RecE exonuclease (PDB 3H4R) and is therefore predicted to function as an exonuclease.

**OG969 Putative glycan-processing enzymes**

Structural similarity searches revealed that OG969 is similar to a glucosyltransferase (PDB: 5X7S), a xyloglucanase (PDB: 6SDU), and an endo-β-N-acetylglucosaminidase A (PDB: 3FHA). These proteins are all involved in glycan modification or degradation (CAZyme), suggesting that OG969 may function as a glycan-processing enzyme.

**OG975 Putative transcriptional regulator**

Structural similarity analysis revealed that OG975 shares structural similarity with PefR, a heme-sensing MarR family transcriptional regulator from *Streptococcus agalactiae* (PDB 7DVT), suggesting that OG975 belongs to the MarR family of transcriptional regulators.

**OG995 FkbM family methyltransferase**

OG995 is structurally similar to FkbM, a methyltransferase from *Methylobacillus flagellatus* KT (PDB 2PY6), suggesting that OG995 may function as a methyltransferase.

**OG999 N-Acetylglucosaminyltransferase**

OG999 is structurally similar to LgtC, a galactosyltransferase from *Neisseria meningitidis* (PDB 1GA8), suggesting that OG999 may function as a glycosyltransferase.

**OG1028 Zinc-binding metallopeptidase**

OG1028 is structurally similar to BACCAC_01431, a putative zinc-binding metallopeptidase from *Bacteroides caccae* (PDB 4L7A). This similarity suggests that OG1028 may function as a zinc-dependent peptidase.

**OG1036 Sodium-dependent citrate symporter (CitS)**

OG1036 is a membrane protein annotated as COG3180 and K07120. Structural similarity analysis revealed similarity to SeCitS, a sodium-dependent citrate symporter from *Salmonella enterica* (PDB: 5A1S), suggesting that OG1036 may function as a membrane transporter involved in the transport of citrate or related carboxylates.

# 4. **Function Unknown (51 OGs)**

**OG171 Uncharacterized membrane protein**

OG171 is an uncharacterized membrane gene annotated as COG3748. No structurally similar proteins were found in the PDB, and no conserved neighboring genes were identified.

**OG311 DUF1178-containing protein**

OG311 is a gene annotated as COG5319 and contains the domain of unknown function DUF1178. No structurally similar proteins or conserved neighboring genes were identified.

**OG327 DUF4167-containing protein**

OG327 is a gene containing the domain of unknown function DUF4167. The predicted structure consists of a hairpin-shaped α-helix flanked by disordered regions, and no structurally similar proteins were identified. An operon including the translation termination factor hemK (53) was conserved in the genomic vicinity.

**OG305 IalB-like membrane-pore protein**

OG305 is a functionally uncharacterized gene. Structural similarity analysis revealed that OG305 shares a monomeric structure similar to IalB, an outer membrane protein of unknown function required for infection of *Bartonella bacilliformis*, a causative agent of cat-scratch disease (54,55). IalB is registered in the PDB as a homo-dodecamer (PDB: 3DTD), suggesting that OG305 may also exist as a multimeric complex. To investigate this, we predicted the dodecameric structure of OG305 using AlphaFold2-Multimer, which produced a complex structure distinct from that of IalB in the PDB (data not shown). Based on the predicted structure and presence of a signal peptide, OG305 is likely to form a pore in the cell membrane as a homo-hexamer or dodecamer. Although the neighboring gene encoding argininosuccinate synthetase (ArgG) was conserved on the SAR11 genomes, its functional relationship to OG305 remains unclear.

**OG251 Membrane protein of the DedA family, potentially associated with aerobic metabolism**

OG251 is annotated as COG0398, a putative membrane protein belonging to the DedA superfamily with unknown function. The DedA superfamily comprises three subfamilies (COG0586, COG0398, and COG1238). COG0398 lacks the conserved residues responsible for the UndP flippase activity found in the functionally characterized DedA subfamily COG0586. It is widely distributed among aerobic bacteria, and gene fusions with genes involved in aerobic metabolism—such as glutathione-thioredoxin reductase and lipoamide dehydrogenase—have been reported (13). COG0398 has been identified as an essential gene in model alphaproteobacteria such as *C. crescentus* and *Dinoroseobacter shibae* (56). In the SAR11 clade, the genomic region surrounding OG251 is conserved and includes the RNase P protein component RnpA.

**OG382 Function unknown periplasmic protein**

OG382 is an uncharacterized gene predicted to encode a protein with a signal peptide. In its genomic vicinity, genes encoding 3-dehydroquinate synthase and shikimate kinase I—components of the Escherichia coli dam superoperon—were found to be conserved (57). No structurally similar proteins have been identified in the PDB.

**OG535 Function unknown protein**

OG535 genomic neighborhood is conserved with OG525 (COG0717, dCTP deaminase) and OG602 (an uncharacterized protein containing a DUF2948 domain) in the SAR11 genomes, although any functional relationship between them remains unclear.

**OG590 Uncharacterized protein**

No structurally similar proteins to OG590 have been registered in the PDB, and its neighboring genes are not conserved. OG590 exhibits a four-helix bundle structure resembling that of the histidine kinase LytSN (58).

**OG602 DUF2948-containing protein**

OG602 is an uncharacterized protein containing a DUF2948 domain. Structural similarity search revealed that its structure has been resolved for a sequence derived from *Neisseria gonorrhoeae* (PDB ID: 5V77); however, no functional information could be obtained. In its genomic neighborhood, dCTP deaminase (Dcd) and other genes of unknown function are conserved.

**OG669 DUF3108 or DUF6134-containing beta-barrel-like protein**

It contains the domains DUF3108 or DUF6134, and predicted structures indicate that it is a beta-barrel protein; however, its function remains unknown.

**OG690 Uncharacterized protein**

OG690 is a functionally uncharacterized gene possessing a signal peptide. The replication initiator gene DnaA is conserved in its genomic neighborhood.

**OG697 DUF3553-containing protein**

OG697 is an uncharacterized gene containing a DUF3553 domain, with no assigned COG or KO annotations. No conserved neighboring genes or structurally similar proteins were identified. The DUF3553 domain is predicted to adopt an SH3 β-barrel fold, and has been suggested to interact with RNA polymerase (59), although the function of this gene remains unknown.

**OG706 DUF2093-containing protein**

OG706 is annotated as COG3791, an uncharacterized conserved protein. It contains a DUF2093 domain, and its genomic neighborhood includes OG446 (COG1560; palmitoleoyl-ACP: Kdo2-lipid-IV acyltransferase LpxP), OG579 (COG0151; phosphoribosylamine–glycine ligase PurD), and OG707 (COG1663; lipid A 4'-kinase LpxK), suggesting a potential role in lipid or purine biosynthesis, though its precise function remains unclear.

**OG730 DUF2794-containing protein**

OG730 is a functionally uncharacterized gene containing the DUF2794 domain. A heat shock protein HspQ gene is conserved in its genomic neighborhood.

**OG733 DUF2065-containing membrane protein**

OG733 is a functionally uncharacterized inner membrane protein that is annotated as K09937 and COG3242. It contains the DUF2065 domain, and its homolog is named YjeT in *E. coli*.

**OG754 DUF6151 super family CENP-V/GFA domain-containing protein**

OG754 is an uncharacterized gene annotated as COG3791 and belongs to the DUF6151 superfamily. It contains a CENP-V/GFA domain and shows structural similarity to an uncharacterized protein of *Rhodobacter sphaeroides* (PDB 3FAC).

**OG798 Function unknown protein PsiE**

OG798 is annotated as K13256 and is predicted to be the phosphate starvation-induced gene PsiE. PsiE is related to a two-component system, but its exact function remains unknown (60,61).

**OG792 DUF2805-containing protein**

OG792 is a functionally uncharacterized gene containing the domain of unknown function DUF2805. No structurally similar proteins are registered in the PDB, and neighboring genes are not conserved.

**OG799 function unknown membrane protein**

OG799 is an uncharacterized gene predicted to encode a protein with transmembrane helices. In its genomic neighborhood, OG569 (COG2137; regulatory protein RecX) and OG907 (uncharacterized) are conserved, but no functional insights could be inferred from these associations.

**OG804 DUF2237-containing protein**

OG804 is annotated as K09966 and COG3651, containing a domain of unknown function DUF2237 (hereafter referred to as DUF2237). It is known that bacteria in the marine *Flavobacteriales* order tend to possess DUF2237 when they carry the gene for the light-driven proton pump proteorhodopsin. Knockout of DUF2237 in *Synechocystis* sp. PCC 6803-P strain results in reduced phototaxis (62). Approximately half of surface marine bacteria utilize light energy via proteorhodopsin, and light-utilization-related genes are crucial for understanding the ecology of marine bacteria (63). Therefore, we conducted a detailed functional analysis of DUF2237, a marine-specific gene related to light utilization.

**OG805 Uncharacterized protein**

OG805 is an uncharacterized gene annotated as COG4274 and contains a proline-binding domain known as GYD (64).

**OG823 DUF502-containing membrane protein**

OG823 was annotated as COG2928 and is a membrane protein gene containing the domain of unknown function DUF502. Approximately 74% of COG2928 sequences were detected from marine environments. DUF502 is a domain found in proteins involved in auxin transport in plants (65).

**OG835 Function unknown protein YqeY**

OG835 was annotated as K09117 and COG1610, corresponding to the yqeY gene. YqeY has been confirmed to be involved in the function of glutaminyl-tRNA synthetase (66), but it is also conserved in species lacking glutaminyl-tRNA synthetase, suggesting that it may have an unknown function (67).

**OG843 TPR-like protein**

OG843 is an uncharacterized gene predicted to encode a protein with a signal peptide. It shows structural similarity to PDB entry 1NA0, suggesting that it may contain a tetratricopeptide repeat (TPR) fold, which is commonly associated with protein–protein interaction.

**OG850 DUF6552-containing membrane protein**

OG850 is a membrane protein of unknown function containing the domain of unknown function DUF6552.

**OG863 DUF2721-containing membrane protein**

OG863 is an uncharacterized membrane protein gene containing a DUF2721 domain. In *Shewanella oneidensis*, point mutations in a membrane protein containing the DUF2721 domain have been reported to confer resistance to formate. However, docking simulations with formate indicate low binding affinity, and the function of this protein remains unknown (68).

**OG872 Uncharacterized membrane protein**

OG872 is a membrane protein gene of unknown function. It contains the low-oxygen-induced mitochondrial protein domain HIG1 (69), but no functional information was obtained.

**OG873 DUF4396-containing membrane protein**

OG873 is a membrane protein gene of unknown function containing the domain of unknown function DUF4396.

**OG875 DUF4864-containing protein**

OG875 is an uncharacterized gene predicted to encode a protein with a signal peptide. It contains a DUF4864 domain, the function of which remains unknown.

**OG880 BMFP Superfamily protein?**

OG880 is an uncharacterized protein group, with most of its sequences lacking COG and KO annotations. However, some sequences within this orthologous group are annotated as CO2960 (ubiquinone biosynthesis accessory factor UbiK), COG5493 (uncharacterized protein containing a PD-(D/E)XK nuclease domain), and COG0172 (seryl-tRNA synthetase), suggesting that OG880 may belong to the BMFP superfamily.

**OG886 Uncharacterized membrane protein**

OG886 is an uncharacterized gene predicted to encode a protein with transmembrane helices.

**OG894 DUF1467 family membrane protein**

OG894 is annotated as COG5454, an uncharacterized DUF1467 family protein found in Alphaproteobacteria.

**OG895 DUF4170 domain-containing protein**

OG895 shows structural similarity to an uncharacterized DUF4170 family protein (PDB: 2MHE). Since only 0.3% of DUF4170 proteins are annotated as belonging to the inositol monophosphatase family in the AnnoDUF database, we classified this OG as functionally uncharacterized.

**OG897 tetratricopeptide repeat-like domain-containing protein**

OG897 is an uncharacterized gene containing a tetratricopeptide repeat (TPR)-like domain. In its genomic neighborhood, OG725 (COG1520; outer membrane protein assembly factor BamB) is conserved, raising the possibility that OG897 may be functionally related to the BAM complex, although further evidence is required.

**OG899 uncharacterized protein (AsmA domain-containing?)**

OG899 is an uncharacterized gene for which no functional annotation could be assigned using either the COG or KO databases. In addition, the surrounding gene context was not conserved, and Foldseek searches did not identify any structurally similar proteins.

**OG904 uncharacterized protein (Potential esterase?)**

OG904 is an uncharacterized gene lacking functional annotation in the COG database. In SAR11 genomes, it is consistently located near OG936 (COG4327, uncharacterized membrane protein containing a DUF4212 domain). Although UniProt and the STRING database annotate OG904 as a potential esterase (e.g., Uniprot Q4FNV2), no structural similarity to known esterases was detected, and the basis for this annotation remains unclear.

**OG907 uncharacterized protein**

OG799 is conserved in the genomic neighborhood of OG907. However, both genes encode uncharacterized membrane proteins, and their functions could not be predicted.

**OG912 YGGT family membrane protein**

OG912 is a membrane protein of approximately 100 amino acids belonging to the YGGT family. In *E. coli*, YggT has been implicated in osmotic regulation, although the underlying mechanism remains unknown (70).

**OG918 Uncharacterized protein**

OG918 consists of very short sequences averaging 64 amino acids, and no functional information was obtained.

**OG928 Uncharacterized membrane protein**

OG928 is a gene of unknown function predicted to contain four transmembrane helices.

**OG934 Uncharacterized membrane protein**

OG934 is a gene of unknown function predicted to contain three transmembrane helices.

**OG936 DUF4212-containing membrane protein**

OG936 is annotated as COG4327 and encodes an uncharacterized protein containing the DUF4212 domain. According to the AnnoDUF database, DUF4212-containing proteins are most frequently annotated as membrane proteins (3%), whereas annotations as RNA polymerase sigma subunits (0.4%) and solute symporter proteins (0.1%) are much less common. These observations suggest that OG936 is likely to be a membrane protein.

**OG941 VanZ-like domain-containing protein**

OG941 is an uncharacterized protein predicted to contain four transmembrane helices. In UniProt, it is annotated as a VanZ-like domain-containing protein. Although VanZ was identified as a resistance factor against cell wall synthesis–inhibiting antibiotics, its precise function remains unknown.

**OG954 DUF4440-containing protein (NTF2 clan, function unknown)**

OG954 is an uncharacterized protein containing the DUF4440 domain. DUF4440 belongs to the nuclear transport factor 2 (NTF2) clan, but its function remains unknown.

**OG1003 A short two-helix protein**

OG1003 is an uncharacterized small protein of up to 75 amino acids in length. Structure prediction suggests that it adopts a hairpin-like structure consisting of two short α-helices.

**OG1013 A short two-helix protein**

OG1013 is an uncharacterized small protein of up to 78 amino acids in length. Structure prediction suggests that it adopts a hairpin-like structure consisting of two short α-helices. OG1013 does not exhibit detectable sequence similarity to OG1003.

**OG1026 SHOCT domain-containing protein**

OG1026 is an uncharacterized protein containing a SHOCT domain. The SHOCT domain is widely distributed among bacteria and is frequently located at the C-terminus of proteins. It has been proposed to be involved in oligomerization and nucleic acid binding; however, its precise biological function remains unknown.

**OG1027 MORN repeat-containing protein**

OG1027 is an uncharacterized protein annotated as COG4642 and containing Membrane Occupation and Recognition Nexus (MORN) repeats. MORN repeats have been identified in animals, plants, and some bacteria; however, their biological function remains unclear.

**OG1048 Zinc-ribbon domain-containing protein**

OG1048 is an uncharacterized protein predicted to contain a zinc-ribbon domain. Although weak structural similarity to a component of the 50S ribosomal subunit from *Borreliella burgdorferi* (PDB 8FN2, probability = 0.92) was detected, the biological function of OG1048 could not be reliably inferred.

**OG1054 Uncharacterized protein**

OG1054 is a small protein of up to 50 amino acids in length, and its biological function remains unknown.

**OG1057 A short two-helix protein**

OG1057 is an uncharacterized small protein of up to 74 amino acids in length and is predicted to adopt a hairpin-like structure formed by two short α-helices.

1.	Daniellou R, Phenix CP, Tam PH, Laliberte MC, Palmer DRJ. Stereoselective oxidation of protected inositol derivatives catalyzed by inositol dehydrogenase from Bacillus subtilis. Org Biomol Chem. 2005 Jan 27;3(3):401–3. doi:10.1039/B417757F

2.	Martens-Uzunova ES, Schaap PJ. An evolutionary conserved d-galacturonic acid metabolic pathway operates across filamentous fungi capable of pectin degradation. Fungal Genet Biol. 2008 Nov 1;45(11):1449–57. doi:10.1016/j.fgb.2008.08.002

3.	Kim SM, Lim HS, Lee SB. Discovery of a RuBisCO-like Protein that Functions as an Oxygenase in the Novel d-Hamamelose Pathway. Biotechnol Bioprocess Eng. 2018 Sep 1;23(5):490–9. doi:10.1007/s12257-018-0305-6

4.	Mukherjee K, Huddleston JP, Narindoshvili T, Nemmara VV, Raushel FM. Functional Characterization of the ycjQRS Gene Cluster from Escherichia coli: A Novel Pathway for the Transformation of d-Gulosides to d-Glucosides. Biochemistry. 2019 Mar 12;58(10):1388–99. doi:10.1021/acs.biochem.8b01278

5.	Rossmann MG, Moras D, Olsen KW. Chemical and biological evolution of a nucleotide-binding protein. Nature. 1974 Jul;250(5463):5463. doi:10.1038/250194a0

6.	Müller GL, Tuttobene M, Altilio M, Martínez Amezaga M, Nguyen M, Cribb P, et al. Light Modulates Metabolic Pathways and Other Novel Physiological Traits in the Human Pathogen Acinetobacter baumannii. J Bacteriol. 2017 Apr 25;199(10):10.1128/jb.00011-17. doi:10.1128/jb.00011-17

7.	Gnanagobal H, Cao T, Hossain A, Vasquez I, Chakraborty S, Chukwu-Osazuwa J, et al. Role of riboflavin biosynthesis gene duplication and transporter in Aeromonas salmonicida virulence in marine teleost fish. Virulence. 2023 Dec 31;14(1):2187025. doi:10.1080/21505594.2023.2187025 PubMed PMID: 36895132.

8.	Steindler L, Schwalbach MS, Smith DP, Chan F, Giovannoni SJ. Energy Starved Candidatus Pelagibacter Ubique Substitutes Light-Mediated ATP Production for Endogenous Carbon Respiration. PLOS ONE. 2011 May 9;6(5):e19725. doi:10.1371/journal.pone.0019725

9.	Reddy UK, Lopez-Ortiz C, Talavera-Caro AG, Natarajan P, Tomason Y, Alaparthi S, et al. GWAS resolves molecular mechanisms underlying natural variation for carotenoids in Cucurbita maxima Duchesne. Sci Hortic. 2023 Mar 15;312:111881. doi:10.1016/j.scienta.2023.111881

10.	Pires RH, Lourenço AI, Morais F, Teixeira M, Xavier AV, Saraiva LM, et al. A novel membrane-bound respiratory complex from Desulfovibrio desulfuricans ATCC 27774. Biochim Biophys Acta BBA - Bioenerg. 2003 Aug 18;1605(1):67–82. doi:10.1016/S0005-2728(03)00065-3

11.	van Vliet DM, von Meijenfeldt FAB, Dutilh BE, Villanueva L, Sinninghe Damsté JS, Stams AJM, et al. The bacterial sulfur cycle in expanding dysoxic and euxinic marine waters. Environ Microbiol. 2021;23(6):2834–57. doi:10.1111/1462-2920.15265

12.	Nguyen HTV, Chen X, Parada C, Luo AC, Shih O, Jeng US, et al. Structure of the heterotrimeric membrane protein complex FtsB-FtsL-FtsQ of the bacterial divisome. Nat Commun. 2023 Apr 5;14(1):1. doi:10.1038/s41467-023-37543-4

13.	Todor H, Herrera N, Gross CA. Three Bacterial DedA Subfamilies with Distinct Functions and Phylogenetic Distribution. mBio. 2023 Mar;14(2):e00028-23. doi:10.1128/mbio.00028-23

14.	Roney IJ, Rudner DZ. The DedA superfamily member PetA is required for the transbilayer distribution of phosphatidylethanolamine in bacterial membranes. Proc Natl Acad Sci. 2023 May 16;120(20):e2301979120. doi:10.1073/pnas.2301979120

15.	Delaby M, Varesio LM, Degeorges L, Crosson S, Viollier PH. The DUF1013 protein TrcR tracks with RNA polymerase to control the bacterial cell cycle and protect against antibiotics. Proc Natl Acad Sci. 2021 Feb 23;118(8):e2010357118. doi:10.1073/pnas.2010357118

16.	McGary K, Nudler E. RNA polymerase and the Ribosome: The Close Relationship. Curr Opin Microbiol. 2013 Apr;16(2):112–7. doi:10.1016/j.mib.2013.01.010 PubMed PMID: 23433801; PubMed Central PMCID: PMC4066815.

17.	Blaha GM, Wade JT. Transcription-Translation Coupling in Bacteria. Annu Rev Genet. 2022;56(1):187–205. doi:10.1146/annurev-genet-072220-033342 PubMed PMID: 36055649.

18.	Bertonati C, Punta M, Fischer M, Yachdav G, Forouhar F, Zhou W, et al. Structural genomics reveals EVE as a new ASCH/PUA-related domain. Proteins Struct Funct Bioinforma. 2009;75(3):760–73. doi:10.1002/prot.22287

19.	Bell RT, Wolf YI, Koonin EV. Modified base-binding EVE and DCD domains: striking diversity of genomic contexts in prokaryotes and predicted involvement in a variety of cellular processes. BMC Biol. 2020 Nov 4;18(1):159. doi:10.1186/s12915-020-00885-2

20.	Mitchell AM, Srikumar T, Silhavy TJ. Cyclic Enterobacterial Common Antigen Maintains the Outer Membrane Permeability Barrier of Escherichia coli in a Manner Controlled by YhdP. mBio. 2018 Aug 7;9(4):10.1128/mbio.01321-18. doi:10.1128/mbio.01321-18

21.	Grimm J, Shi H, Wang W, Mitchell AM, Wingreen NS, Huang KC, et al. The inner membrane protein YhdP modulates the rate of anterograde phospholipid flow in Escherichia coli. Proc Natl Acad Sci. 2020 Oct 27;117(43):26907–14. doi:10.1073/pnas.2015556117

22.	Cooper BF, Clark R, Kudhail A, Bhabha G, Ekiert DC, Khalid S, et al. Phospholipid transport to the bacterial outer membrane through an envelope-spanning bridge. BioRxiv Prepr Serv Biol. 2023 Oct;2023.10.05.561070. doi:10.1101/2023.10.05.561070 PubMed PMID: 37873249; PubMed Central PMCID: PMC10592960.

23.	Anwari K, Webb CT, Poggio S, Perry AJ, Belousoff M, Celik N, et al. The evolution of new lipoprotein subunits of the bacterial outer membrane BAM complex. Mol Microbiol. 2012;84(5):832–44. doi:10.1111/j.1365-2958.2012.08059.x

24.	Ginalski K, Kinch L, Rychlewski L, Grishin NV. DCC proteins: a novel family of thiol-disulfide oxidoreductases. Trends Biochem Sci. 2004 Jul 1;29(7):339–42. doi:10.1016/j.tibs.2004.04.003 PubMed PMID: 15236740.

25.	Milano T, Angelaccio S, Tramonti A, di Salvo ML, Nogues I, Contestabile R, et al. A Comprehensive Computational Analysis of Mycobacterium Genomes Pinpoints the Genes Co-occurring with YczE, a Membrane Protein Coding Gene Under the Putative Control of a MocR, and Predicts its Function. Interdiscip Sci Comput Life Sci. 2018 Mar 1;10(1):111–25. doi:10.1007/s12539-017-0266-z

26.	Worley JN, Russell AB, Wexler AG, Bronstein PA, Kvitko BH, Krasnoff SB, et al. Pseudomonas syringae pv. tomato DC3000 CmaL (PSPTO4723), a DUF1330 Family Member, Is Needed To Produce l-allo-Isoleucine, a Precursor for the Phytotoxin Coronatine. J Bacteriol. 2013 Dec 26;195(2):287–96. doi:10.1128/jb.01352-12

27.	Grocholski T, Koskiniemi H, Lindqvist Y, Mäntsälä P, Niemi J, Schneider G. Crystal Structure of the Cofactor-Independent Monooxygenase SnoaB from Streptomyces nogalater: Implications for the Reaction Mechanism. Biochemistry. 2010 Feb 9;49(5):934–44. doi:10.1021/bi901985b

28.	Morton CJ, Campbell ID. SH3 Domains: Molecular ‘Velcro’1. Curr Biol. 1994 Jul 1;4(7):615–7. doi:10.1016/S0960-9822(00)00134-2

29.	Thoma R, Löffler B, Stihle M, Huber W, Ruf A, Hennig M. Structural basis of proline-specific exopeptidase activity as observed in human dipeptidyl peptidase-IV. Struct Lond Engl 1993. 2003 Aug;11(8):947–59. doi:10.1016/s0969-2126(03)00160-6 PubMed PMID: 12906826.

30.	Wells RM, Jones CM, Xi Z, Speer A, Danilchanka O, Doornbos KS, et al. Discovery of a Siderophore Export System Essential for Virulence of Mycobacterium tuberculosis. PLOS Pathog. 2013 Jan 31;9(1):e1003120. doi:10.1371/journal.ppat.1003120

31.	Nikaido H, Takatsuka Y. Mechanisms of RND multidrug efflux pumps. Biochim Biophys Acta BBA - Proteins Proteomics. 2009 May 1;Mechanisms of Drug Efflux and Strategies to Combat Them1794(5):769–81. doi:10.1016/j.bbapap.2008.10.004

32.	Weaver AI, Alvarez L, Rosch KM, Ahmed A, Wang GS, van Nieuwenhze MS, et al. Lytic transglycosylases mitigate periplasmic crowding by degrading soluble cell wall turnover products. Kana BD, Storz G, Garner E, editors. eLife. 2022 Jan 24;11:e73178. doi:10.7554/eLife.73178

33.	de la Mora J, Ballado T, González-Pedrajo B, Camarena L, Dreyfus G. The Flagellar Muramidase from the Photosynthetic Bacterium Rhodobacter sphaeroides. J Bacteriol. 2007 Nov 15;189(22):7998–8004. doi:10.1128/jb.01073-07

34.	Buttigieg PL, Hankeln W, Kostadinov I, Kottmann R, Yilmaz P, Duhaime MB, et al. Ecogenomic Perspectives on Domains of Unknown Function: Correlation-Based Exploration of Marine Metagenomes. PLOS ONE. 2013 Mar 14;8(3):e50869. doi:10.1371/journal.pone.0050869

35.	Holmquist M. Alpha/Beta-hydrolase fold enzymes: structures, functions and mechanisms. Curr Protein Pept Sci. 2000 Sep;1(2):209–35. doi:10.2174/1389203003381405 PubMed PMID: 12369917.

36.	Varadi M, Anyango S, Deshpande M, Nair S, Natassia C, Yordanova G, et al. AlphaFold Protein Structure Database: massively expanding the structural coverage of protein-sequence space with high-accuracy models. Nucleic Acids Res. 2022 Jan 7;50(D1):D439–44. doi:10.1093/nar/gkab1061

37.	Bergé M, Campagne S, Mignolet J, Holden S, Théraulaz L, Manley S, et al. Modularity and determinants of a (bi-)polarization control system from free-living and obligate intracellular bacteria. Veening JW, editor. eLife. 2016 Dec 23;5:e20640. doi:10.7554/eLife.20640

38.	Mignolet J, Holden S, Bergé M, Panis G, Eroglu E, Théraulaz L, et al. Functional dichotomy and distinct nanoscale assemblies of a cell cycle-controlled bipolar zinc-finger regulator. Mignot T, editor. eLife. 2016 Dec 23;5:e18647. doi:10.7554/eLife.18647

39.	Lu N, Duvall SW, Zhao G, Kowallis KA, Zhang C, Tan W, et al. Scaffold-Scaffold Interaction Facilitates Cell Polarity Development in Caulobacter crescentus. mBio. 2023 Mar 27;14(2):e03218-22. doi:10.1128/mbio.03218-22

40.	Horita S, Scotti JS, Thinnes C, Mottaghi-Taromsari YS, Thalhammer A, Ge W, et al. Structure of the Ribosomal Oxygenase OGFOD1 Provides Insights into the Regio- and Stereoselectivity of Prolyl Hydroxylases. Structure. 2015 Apr 7;23(4):639–52. doi:10.1016/j.str.2015.01.014

41.	Scotti JS, Leung IKH, Ge W, Bentley MA, Paps J, Kramer HB, et al. Human oxygen sensing may have origins in prokaryotic elongation factor Tu prolyl-hydroxylation. Proc Natl Acad Sci. 2014 Sep 16;111(37):13331–6. doi:10.1073/pnas.1409916111

42.	Bouvier J, Pugsley AP, Stragier P. A gene for a new lipoprotein in the dapA-purC interval of the Escherichia coli chromosome. J Bacteriol. 1991 Sep;173(17):5523–31. doi:10.1128/jb.173.17.5523-5531.1991 PubMed PMID: 1885529; PubMed Central PMCID: PMC208266.

43.	Hocher A, Laursen SP, Radford P, Tyson J, Lambert C, Stevens KM, et al. Histones with an unconventional DNA-binding mode in vitro are major chromatin constituents in the bacterium Bdellovibrio bacteriovorus. Nat Microbiol. 2023 Nov;8(11):11. doi:10.1038/s41564-023-01492-x

44.	Hashimoto W, Maruyama Y, Nakamichi Y, Mikami B, Murata K. Crystal Structure of Pedobacter heparinus Heparin Lyase Hep III with the Active Site in a Deep Cleft. Biochemistry. 2014 Feb 4;53(4):777–86. doi:10.1021/bi4012463

45.	Tsilibaris V, Maenhaut-Michel G, Van Melderen L. Biological roles of the Lon ATP-dependent protease. Res Microbiol. 2006 Oct 1;157(8):701–13. doi:10.1016/j.resmic.2006.05.004

46.	Brézellec P, Vallet-Gely I, Possoz C, Quevillon-Cheruel S, Ferat JL. DciA is an ancestral replicative helicase operator essential for bacterial replication initiation. Nat Commun. 2016 Nov 10;7(1):1. doi:10.1038/ncomms13271

47.	Ozaki S, Wang D, Wakasugi Y, Itani N, Katayama T. The Caulobacter crescentus DciA promotes chromosome replication through topological loading of the DnaB replicative helicase at replication forks. Nucleic Acids Res. 2022 Dec 9;50(22):12896–912. doi:10.1093/nar/gkac1146

48.	Bitto E, McKay DB. Crystallographic Structure of SurA, a Molecular Chaperone that Facilitates Folding of Outer Membrane Porins. Structure. 2002 Nov 1;10(11):1489–98. doi:10.1016/S0969-2126(02)00877-8

49.	Buddelmeijer N, Judson N, Boyd D, Mekalanos JJ, Beckwith J. YgbQ, a cell division protein in Escherichia coli and Vibrio cholerae, localizes in codependent fashion with FtsL to the division site. Proc Natl Acad Sci U S A. 2002 Apr;99(9):6316–21. doi:10.1073/pnas.092128499 PubMed PMID: 11972052; PubMed Central PMCID: PMC122946.

50.	Ndi M, Masuyer G, Dawitz H, Carlström A, Michel M, Elofsson A, et al. Structural basis for the interaction of the chaperone Cbp3 with newly synthesized cytochrome b during mitochondrial respiratory chain assembly. J Biol Chem. 2019 Nov 8;294(45):16663–71. doi:10.1074/jbc.RA119.010483

51.	McNeil MB, Clulow JS, Wilf NM, Salmond GPC, Fineran PC. SdhE Is a Conserved Protein Required for Flavinylation of Succinate Dehydrogenase in Bacteria*. J Biol Chem. 2012 May 25;287(22):18418–28. doi:10.1074/jbc.M111.293803

52.	Grill B, Pavkov-Keller T, Grininger C, Darnhofer B, Gruber K, Hall M, et al. Engineering TM1459 for Stabilisation against Inactivation by Amino Acid Oxidation. Chem Ing Tech. 2023;95(4):596–606. doi:10.1002/cite.202200176

53.	Nakahigashi K, Kubo N, Narita S ichiro, Shimaoka T, Goto S, Oshima T, et al. HemK, a class of protein methyl transferase with similarity to DNA methyl transferases, methylates polypeptide chain release factors, and hemK knockout induces defects in translational termination. Proc Natl Acad Sci. 2002 Feb 5;99(3):1473–8. doi:10.1073/pnas.032488499

54.	Coleman SA, Minnick MF. Establishing a Direct Role for the Bartonella bacilliformis Invasion-Associated Locus B (IalB) Protein in Human Erythrocyte Parasitism. Infect Immun. 2001 Jul;69(7):4373–81. doi:10.1128/iai.69.7.4373-4381.2001

55.	Deng H, Pang Q, Xia H, Le Rhun D, Le Naour E, Yang C, et al. Identification and functional analysis of invasion associated locus B (IalB) in Bartonella species. Microb Pathog. 2016 Sep 1;98:171–7. doi:10.1016/j.micpath.2016.05.007

56.	Price MN, Wetmore KM, Waters RJ, Callaghan M, Ray J, Liu H, et al. Mutant phenotypes for thousands of bacterial genes of unknown function. Nature. 2018 May;557(7706):7706. doi:10.1038/s41586-018-0124-0

57.	Lyngstadaas A, Løbner-Olesen A, Grelland E, Boye E. The gene for 2-phosphoglycolate phosphatase (gph) in Escherichia coli is located in the same operon as dam and at least five other diverse genes. Biochim Biophys Acta BBA - Gen Subj. 1999 Oct 18;1472(1):376–84. doi:10.1016/S0304-4165(99)00146-4

58.	Li J, Wang C, Yang G, Sun Z, Guo H, Shao K, et al. Molecular mechanism of environmental d-xylose perception by a XylFII-LytS complex in bacteria. Proc Natl Acad Sci. 2017 Aug;114(31):8235–40. doi:10.1073/pnas.1620183114

59.	Timinskas K, Venclovas Č. New insights into the structures and interactions of bacterial Y-family DNA polymerases. Nucleic Acids Res. 2019 May 21;47(9):4393–405. doi:10.1093/nar/gkz198

60.	Kim SK, Kimura S, Shinagawa H, Nakata A, Lee KS, Wanner BL, et al. Dual Transcriptional Regulation of theEscherichia coli Phosphate-Starvation-InduciblepsiE Gene of the Phosphate Regulon by PhoB and the Cyclic AMP (cAMP)-cAMP Receptor Protein Complex. J Bacteriol. 2000 Oct;182(19):5596–9. doi:10.1128/jb.182.19.5596-5599.2000

61.	Zhou L, Lei XH, Bochner BR, Wanner BL. Phenotype MicroArray Analysis of Escherichia coli K-12 Mutants with Deletions of All Two-Component Systems. J Bacteriol. 2003 Aug 15;185(16):4956–72. doi:10.1128/jb.185.16.4956-4972.2003

62.	Kumagai Y, Yoshizawa S, Nakajima Y, Watanabe M, Fukunaga T, Ogura Y, et al. Solar-panel and parasol strategies shape the proteorhodopsin distribution pattern in marine Flavobacteriia. ISME J. 2018 May;12(5):5. doi:10.1038/s41396-018-0058-4

63.	Finkel OM, Béjà O, Belkin S. Global abundance of microbial rhodopsins. ISME J. 2013 Feb;7(2):2. doi:10.1038/ismej.2012.112

64.	Freund C, Dötsch V, Nishizawa K, Reinherz EL, Wagner G. The GYF domain is a novel structural fold that is involved in lymphoid signaling through proline-rich sequences. Nat Struct Biol. 1999 Jul;6(7):656–60. doi:10.1038/10712 PubMed PMID: 10404223.

65.	Liu F, Zhang L, Luo Y, Xu M, Fan Y, Wang L. Interactions of Oryza sativa OsCONTINUOUS VASCULAR RING-LIKE 1 (OsCOLE1) and OsCOLE1-INTERACTING PROTEIN reveal a novel intracellular auxin transport mechanism. New Phytol. 2016;212(1):96–107. doi:10.1111/nph.14021

66.	Hadd A, Perona JJ. Coevolution of Specificity Determinants in Eukaryotic Glutamyl- and Glutaminyl-tRNA Synthetases. J Mol Biol. 2014 Oct 23;426(21):3619–33. doi:10.1016/j.jmb.2014.08.006

67.	Deniziak M, Sauter C, Becker HD, Paulus CA, Giegé R, Kern D. Deinococcus glutaminyl-tRNA synthetase is a chimer between proteins from an ancient and the modern pathways of aminoacyl-tRNA formation. Nucleic Acids Res. 2007 Mar 1;35(5):1421–31. doi:10.1093/nar/gkl1164

68.	Cross MCG, Aboulnaga E, TerAvest MA. A small number of point mutations confer formate tolerance in Shewanella oneidensis. Appl Environ Microbiol. 2025 Apr 10;91(5):e01968-24. doi:10.1128/aem.01968-24

69.	Wang J, Cao Y, Chen Y, Chen Y, Gardner P, Steiner DF. Pancreatic β cells lack a low glucose and O2-inducible mitochondrial protein that augments cell survival. Proc Natl Acad Sci. 2006 Jul 11;103(28):10636–41. doi:10.1073/pnas.0604194103

70.	ITO T, UOZUMI N, NAKAMURA T, TAKAYAMA S, MATSUDA N, AIBA H, et al. The Implication of YggT of Escherichia coli in Osmotic Regulation. Biosci Biotechnol Biochem. 2009 Dec 23;73(12):2698–704. doi:10.1271/bbb.90558
